# Regional language effects on accent perception and language attitude: The case of mandarin vs. cantonese speakers in mainland China

**DOI:** 10.1371/journal.pone.0352330

**Published:** 2026-07-06

**Authors:** Yizhou Lan, Jeroen van de Weijer, Albert Lee

**Affiliations:** 1 College of International Studies, Shenzhen University, Shenzhen, China; 2 Department of Linguistics and Modern Languages, The Education University of Hong Kong, Tai Po, Hong KongChina; University of Missouri Columbia, UNITED STATES OF AMERICA

## Abstract

The perception of L2 English accents has been extensively studied across disparate language groups, yet it remains unclear how these judgments operate among speakers of closely related varieties, where shared linguistic heritage might heighten sensitivity to phonetic differences. This study investigates how L2 English accent judgments operate among L2 English speakers whose L1s belong to the same broader language family. We recruited 164 English learners in mainland China with Cantonese and Mandarin backgrounds defined here by their first-acquired and dominant spoken Chinese variety (i.e., L1/dominant Cantonese vs. L1/dominant Mandarin), and asked them to rate the comprehensibility and accentedness of 12 pre-categorized Mandarin- and Cantonese-accented English short passages of different accent strengths and to rate 18 attitudinal traits concerning the superiority, attractiveness and dynamism of those same short passages. Two native English short passages were also added as controls. Results show that the native English speakers were rated less accented than and socially superior to both Mandarin- and Cantonese-accented English regardless of accent strengths. Both Cantonese and Mandarin speakers tended to rate Cantonese-accented English as more accented than Mandarin-accented ones, although both had similar level of comprehensibility. In terms of language attitude, Cantonese-accented English was rated as more friendly but lower-class compared to Mandarin-accented English in some aspects, whereas Mandarin-accented English was rated as more dynamic than and superior to Cantonese-accented English by both L1 Cantonese and Mandarin speakers. Results show that both Cantonese and Mandarin speakers of English considered native English as the superior variant of accent.

## Introduction

Cantonese, Putonghua (Mandarin), and English are the main languages used in the Guangdong–Hong Kong–Macao Greater Bay Area (GBA), an economic and innovation hub in southeast China. As Cantonese is an official language only in Hong Kong, but not in the Guangdong province, this divergence between *de jure* policy and *de facto* language use provides a useful context for examining the sociolinguistic structuring of language attitudes. Earlier studies (e.g., [1]) investigated Hong Kong English learners’ perceptions of Cantonese, English and Mandarin and found that Cantonese was strongly associated with local identity, English with power and socioeconomic mobility, and Putonghua with increasing instrumental value, suggesting its potential rise in symbolic prestige, understood here as the attribution of social authority and capital rather than official recognition [2,3]. Earlier matched-guise studies likewise showed a functional differentiation between English and Cantonese, with English indexing power and modernity and Cantonese solidarity and intimacy [4,5]. More recent work reports increased affective acceptance of localized English alongside stronger orientations toward local identity [6], consistent with broader evidence of growing valorization of local linguistic resources in Hong Kong [7].

On the other hand, the language attitudes of Cantonese speakers in the Guangdong province of Mainland China remain relatively understudied. We have seen an improvement in the status of standard Mandarin (*aka* Putonghua) in Guangdong, which was lower than that of Cantonese back in the 1990s [8], but has since risen to a higher level than the regional language [9]. Ng and Zhao [10] found that the importance of Cantonese is being gradually replaced by Mandarin in terms of the number of speakers and that Cantonese has been losing national and international prestige in their perception. While native speakers of Cantonese hold on to the identity of Cantonese family, socialization, friendliness and closeness through its use, Mandarin is considered the standard language and a sine qua non to advance education and career, securing its instrumental prestige. The migration of many Mandarin speakers from northern China has also accelerated the use of Mandarin in Guangdong [11,12].

According to our pilot survey [Unpublished] (N = 112), 56% of Guangdong speakers of Cantonese self-reported to have acquired Cantonese before Mandarin, whereas the rest reported to pick up the two languages simultaneously. As a result, most Guangdong learners of English use Cantonese as their first language (L1), Mandarin as their second language (L2), and English as their third language (L3) (CME henceforth). As Murphy [13] argued, the learning transfer from L1 and L2 to L3 can be seen as a complicated procedure in which the L1 and L2 can affect L3 acquisition either as a whole or on their own [14–16]. Chen and Han [14] found that Cantonese speakers (CEM) from Hong Kong tend to make pronunciation errors in their L2 English and L3 Mandarin which can be predicted from the phonological differences between their L2 or L3 and L1 Cantonese. The next natural question would thus be whether the order of language acquisition may be associated with variation in language attitude.

The evaluation of a second-language accent is often approached from three core perceptual dimensions—intelligibility (actual degree of understanding), comprehensibility (perceived ease), and accentedness (strength of accent) [17]—although a wide range of additional social, ideological, and affective factors have also been examined in the broader language attitudes literature [18,19]. A listener’s bias towards a certain type of accent is shown when high intelligibility and high accentedness ratings co-occur [20]. Such bias can be a result of social factors such as perceived superiority [21], educatedness and competence [22], and friendliness and attractiveness [23,24] of the speaker, which are key components of a listener’s language attitude. Previous studies on Japanese learners showed that Japanese-accented English is perceived as friendlier and preferred by Japanese learners of English [25]. However, Mandarin Chinese English learners are believed to discriminate against their own L2-colored English accents in terms of stereotyping and prejudicing in academic and career-oriented settings [26,27]. Recent work has also shown that both linguistic properties and social expectations jointly shape L2 speech perception. For example, listeners’ judgments of intelligibility, comprehensibility, and accentedness can be influenced not only by prosodic accuracy but also by perceived speaker ethnicity, even when the acoustic signal remains constant [28].

Therefore, the present study primarily examines how listeners’ dominant linguistic background (Cantonese vs. Mandarin) shapes their perception of L2 English accents and associated language attitudes in the multilingual context of the Greater Bay Area. In particular, we ask whether Cantonese- and Mandarin-dominant listeners differ systematically in their evaluations of Mandarin- and Cantonese-accented English across dimensions of intelligibility, comprehensibility, accentedness, and social traits. In addition, we explore whether patterns of language acquisition (e.g., CME, MCE, MEC) are associated with variation in these perceptual and attitudinal judgments. However, given the uneven distribution of acquisition-order subgroups, this aspect of the analysis is treated as exploratory.

By addressing these questions, the study aims to clarify how closely related linguistic backgrounds interact with accent perception and language attitudes in a rapidly changing sociolinguistic environment.

## Materials and Methods

### Participants

A total of 164 English-majoring college students from Guangdong participated in (i) an accent perception task and (ii) an accent trait identification task. Participants’ dominant language background was self-reported and cross-checked through an online background questionnaire. Among them, 70 were trilingual speakers. Data from 15 participants (9.1%) were subsequently discarded as they were later found to come from irrelevant L1 backgrounds (e.g., L1 Russian). Of the remaining 149 participants, 45 acquired Cantonese, then Mandarin and English (CME), 14 were MCE, 7 were MEC, 2 were CEM, and 81 only spoke Mandarin and English (ME) without any noticeable accent from other regional Chinese languages, based on the judgment by an expert phonetician who speaks Mandarin and English. However, we do acknowledge that participants classified as Mandarin-dominant may still have had varying degrees of passive exposure to Cantonese, which is pervasive in the regional linguistic environment. We made sure that the Cantonese speakers were all descendants of monolingual Cantonese-speaking parents. English was the only foreign language studied by all participants, who had studied it for an average of 11.8 years. Their English scores on the National College Entrance Examination ranged between 95 and 141 out of 150 with an average of 125.2, so they could be collectively described as advanced learners of English. Among the 149 participants included in the analysis, 131 were identified as female and 18 as male. Gender was not included as a predictor in the statistical models, as it was not a primary focus of the study.

### Stimuli and Procedure

Language attitude is often studied with a matched-guise test (MGT), in which listeners are asked to rate a speech sample with different accents spoken by a single speaker who is proficient in those accents. However, one limitation of this method is that the talker may not be equally proficient in all the varieties concerned. Therefore, here we used spoken short passages of different accent strengths, which were categorized by expert linguists as rating materials. The materials for both the accent perception task and the language attitude task were 14 pre-recorded short passages downloadable from the Speech Accent Archive [29] (Accessed in July 2023). The stimulus passage is presented as follows.

“*Please call Stella. Ask her to bring these things with her from the store: Six spoons of fresh snow peas, five thick slabs of blue cheese, and maybe a snack for her brother Bob. We also need a small plastic snake and a big toy frog for the kids. She can scoop these things into three red bags, and we will go meet her Wednesday at the train station.*”

This passage from Speech Accent Archive [29] is a standardized elicitation text widely used in accent and pronunciation research. Although its content may appear unusual in everyday discourse, it is intentionally constructed to elicit a wide range of segmental and suprasegmental features (e.g., consonant clusters, vowel contrasts, stress patterns, and prosody) in a controlled and comparable manner across speakers. We used these stimuli to prioritize cross-speaker comparability over naturalness, and the reason for doing so is to ensure tighter phonetic control of the experimental setting, [30,31], although we do acknowledge that listeners may base their ratings on paralinguistic components of the speaker’s voice, which was a limitation unable to mitigate while the study was designed.

The recordings of these short passages were used in its original form download from the archive and then categorized by five phonetically trained native English-speaking judges who are fluent in Cantonese and Mandarin into three levels of strength: strong-accent (H), moderate-accent (M), and weak-accent (L). As a result, the talkers of the passages were categorized into (i) twelve Mandarin and Cantonese speakers of English categorized as having strong, moderate, or weak accents and (ii) two Standard American English speakers (see Table 1 for number of recordings and Speaker IDs and other metadata of the recordings available on the Archive). The phoneticians’ judgments were based on overall perceived accentedness, defined as the degree of deviation from a native-speaker norm. The judges agreed on the categorization of all 12 talkers (Cronbach’s *α* = .828). All talker identities were concealed and not provided for the participants. In subsequent statistical analyses, the original “talker” variable, which had 14 levels corresponding to individual talkers, was recoded into a categorical factor with 7 levels representing distinct accent strength conditions (see Table 1). This recoding reflected assigned accent strength rather than individual identity, so the factor was effectively modeled as “accent strength” in the analysis. Although no explicit normalization procedures were applied, the recordings were produced under relatively controlled conditions and presented to all participants using the same playback equipment. We acknowledge that residual variation in amplitude and duration may introduce some variability in perception, and this is considered a limitation of the study.

**Table 1:** Sociodemographic characteristics of talkers by language variety and accent.

Language Variety	Accent Strength	Talker ID (SAA)	Gender	Age	Birthplace	L1 Background
Cantonese	Weak	Cantonese No. 9	M	22	Hong Kong	Cantonese
Cantonese	Weak	Cantonese No. 17	F	26	Hong Kong	Cantonese
Cantonese	Moderate	Cantonese No. 3	M	22	Hong Kong	Cantonese
Cantonese	Moderate	Cantonese No. 5	M	18	Hong Kong	Cantonese
Cantonese	Strong	Cantonese No. 1	F	22	Hong Kong	Cantonese
Cantonese	Strong	Cantonese No. 2	M	20	Hong Kong	Cantonese
Mandarin	Weak	Mandarin No. 33	M	25	Beijing	Mandarin
Mandarin	Weak	Mandarin No. 87	F	22	Gansu	Mandarin
Mandarin	Moderate	Mandarin No. 118	F	24	Shaanxi	Mandarin
Mandarin	Moderate	Mandarin No. 139	M	23	Yunnan	Mandarin
Mandarin	Strong	Mandarin No. 113	M	29	Inner Mongolia	Mandarin
Mandarin	Strong	Mandarin No. 114	F	19	Beijing	Mandarin
English (control)	—	American English No. 33	M	22	San Diego, CA, USA	English
English (control)	—	American English No. 123	F	28	Riverside, CA, USA	English

In forming these judgments, the phoneticians listened to these excerpts and recorded recurrent segmental and suprasegmental features commonly reported for Mandarin- and Cantonese-accented English, including consonantal substitutions (e.g.,/θ/→[s],/r/–/l/ variation), vowel quality differences, syllable-timing tendencies, deletion or epenthesis preferences, and prosodic transfer such as reduced stress contrast and intonational patterns [30–32]. Eventually, judges rated accentedness on a 3-point scale (weak, moderate, strong).

In the accent rating task, participants were seated in a computer room with HD headphones and listened to the short passages. First, participants provided language background information such as gender, age, gaokao English score, IELTS score (if available), dominant language background, the order of acquisition of regional languages (Cantonese and Mandarin), etc. on an online survey system (www.wjx.com). Dominant language background was self-reported and cross-checked. Then they were instructed to provide intelligibility, comprehensibility, and perceived accentedness scores on the following language attitude evaluation sheet. All scores were obtained through the same system. The participants provided intelligibility scores by completing a comprehension test (on a scale of 0–4, where 4 indicated that four correct answers to the four questions were provided, randomly selected from the following: “Who was called? What was bought from the store? What was bought for Bob? What were bought for the kids? How many red bags were there? When were they going to meet? Where were they going to meet?”). Each question contained four choices, only one of which was correct. Scores were linearly rescaled from 0–4–1–5 to align with the other scores. Participants also gave comprehensibility and accentedness scores by rating statements such as “I don’t have to make an effort to understand the short passage,” and “the short passage is heavily accented,” respectively, on a Likert scale from 1 (strongly disagree) to 5 (strongly agree). All three indicators were subsequently aligned to a 1–5 scale. The questionnaire was in Chinese and then translated into English in the Supporting Information 1

It should be noted that all talkers produced the same elicitation passage. While this allows for controlled comparison across speakers, it may introduce familiarity or carryover effects, whereby listeners’ comprehension of later stimuli is facilitated by prior exposure to the content. As a result, intelligibility scores in the present study were not analyzed in the results. Immediately following the accent rating task, participants were asked to listen to the same 14 talkers’ Cantonese-, Mandarin- and native-accented English productions in random order, and to make attitudinal judgments of these short passages on 18 attitudinal traits in individual adjectives (see S1 File). They include 6 ***superiority*** traits: intelligent, educated, competent, rich, blue-collar, and experienced; 6 ***attractiveness*** traits: friendly, arrogant, sincere, approachable, considerate, and trustworthy; and 6 ***dynamism*** traits: industrious, aggressive, trendy, passive, shy, and confident. Ratings were based on a 5-point Likert scale (1 = strongly disagree, 5 = strongly agree). To avoid potential terminological ambiguity, the category labelled here as ***superiority*** largely corresponds to what is more commonly referred to as “status and competence” in the language attitudes literature, encompassing evaluations related to education, intelligence, occupational standing, and expertise (e.g., [19,23]). The 18 attitudinal traits were adapted from Lai [1], a questionnaire designed for a similar purpose and especially for Cantonese speakers. At the same time, we acknowledge that frameworks such as Zahn and Hopper’s [24] tripartite distinction (superiority, attractiveness, dynamism) are not fully cross-culturally validated, and that affective meaning dimensions may vary across sociocultural settings [33]. In the present study, these categories are therefore treated as heuristic organizing dimensions rather than fixed psychological universals. Since Lai’s [1] questionnaire was not evenly balanced across the three evaluative dimensions, we supplemented it with additional traits to ensure comparable representation of superiority, attractiveness, and dynamism. These additional items were selected based on three considerations: (i) their frequent use in prior language attitude research (e.g., [24,25]), (ii) their relevance to dimensions of social evaluation such as competence, solidarity, and agency, and (iii) their perceived salience in the present sociolinguistic context, as indicated by pilot testing with a comparable participant group (N = 112). While these traits are theoretically motivated and contextually informed, we do not assume that they exhaustively capture all culturally specific evaluative dimensions; rather, they provide a structured basis for comparative analysis across accent conditions. (See S1–S2 File). To avoid positive bias, 5 of the 18 items were deliberately designed as negative traits (marked with an asterisk), and the statistical analyses of these items were accounted for in reverse. The remaining 13 items were positive traits.

The accent rating and the attitude rating tasks were carried out and completed on 18 July 2023 in a computerized classroom in a university in Guangdong, China. Before both tasks, the 164 participants provided written informed consent that they would like to voluntarily participate in this research. This study falls out of the scope of obtaining an ethics review in the authors’ institutions and thus does not require IRB approval based on regulations in the authors’ geographic locations.

### Data analysis

As the distribution of acquisition-order subgroups was uneven, and therefore analyses involving acquisition order should be interpreted as exploratory. A principal component analysis (PCA) was conducted on 20 items using oblique (direct oblimin) rotation. The Kaiser-Meyer-Olkin measure verified sampling adequacy for the analysis, KMO = .932, indicating excellent data suitability. Bartlett’s test of sphericity, χ²(190) = 26,064.94, *p* < .001, confirmed that correlations between items were sufficiently large for PCA. An initial analysis identified three components with eigenvalues exceeding Kaiser’s criterion of 1, collectively explaining 65.60% of the variance. To facilitate comparison with prior analyses and to align with theoretical constructs, we specified extraction of four PCs, which yielded four components with eigenvalues over Kaiser’s criterion of 0.949, explaining a cumulative 66.55% of the variance. Supporting Information 3 presents the rotated factor loadings for this four-component solution. The item loadings suggest that: component 1 (PC1) primarily captures superiority traits, component 2 (PC2) mainly attractiveness traits, component 3 (PC3) dynamism traits, and component 4 (PC4) accent-related measurements.

We fitted linear mixed-effects models to the factor scores of the four principal components using the R package *lmerTest* [34]. Model selection was performed via a bottom-up approach, guided by likelihood ratio tests using the *anova()* function. The initial (baseline) model included only random intercepts for each participant (“Subject”), with no fixed effects. After that, we added the fixed effect of talker (in turn, accent strength). Sequentially, fixed effects for demographic factors were added one at a time in the following order: sex, age, L1, number of years studying English, order of acquisition, *Gaokao* English score, IELTS band score (if available), and years speaking Cantonese and Mandarin. At each step, the inclusion of a fixed factor was retained only if it resulted in a statistically significant improvement in model fit, as determined by the likelihood ratio test (*p* < .05). None of the demographic factors contributed significantly to model fit and were therefore excluded from the final models. Finally, including by-participant random slopes for talker was attempted but resulted in overparameterization. The final models thus included only the random intercept for participant and the fixed effect of talker.

For comprehensiveness’ sake (see Supporting Information 2), we also fitted cumulative link mixed-effects models to the 20 individual trait scores (2 accent evaluation and 18 language attitude traits) of scaled results using the R package ordinal [35]. This allows us to directly see the effect of talker (in turn accent strength) on individual trait scores before PCA was run.

## Results

**Fig 1:**
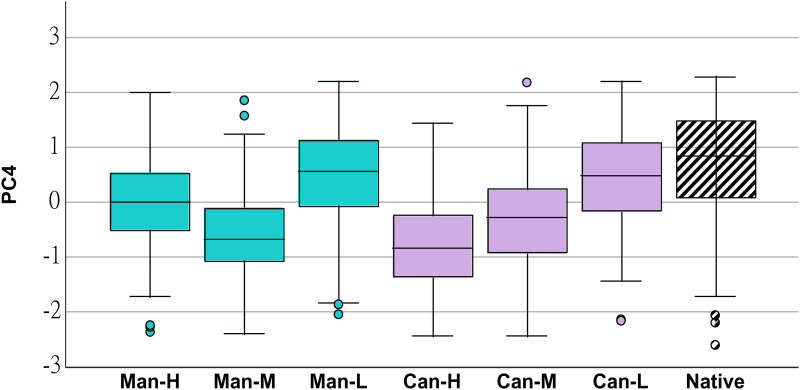
Summary of PC4 scores by accent strength. PC3 scores across accent strength conditions.

**Fig 2:**
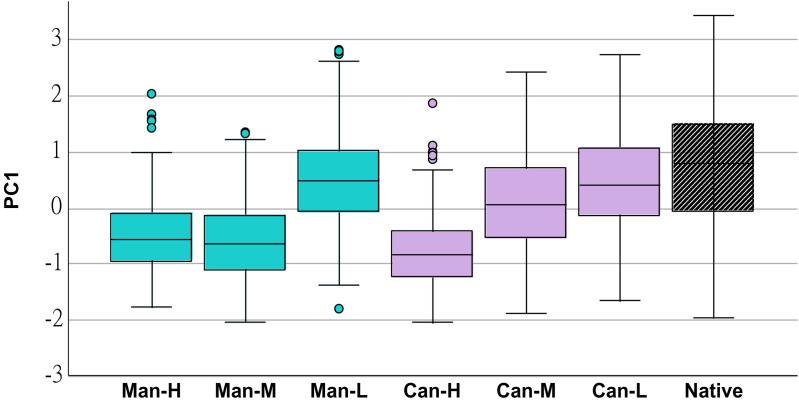
Summary of PC1 scores by accent strength. PC1 scores across accent strength conditions.

**Fig 3:**
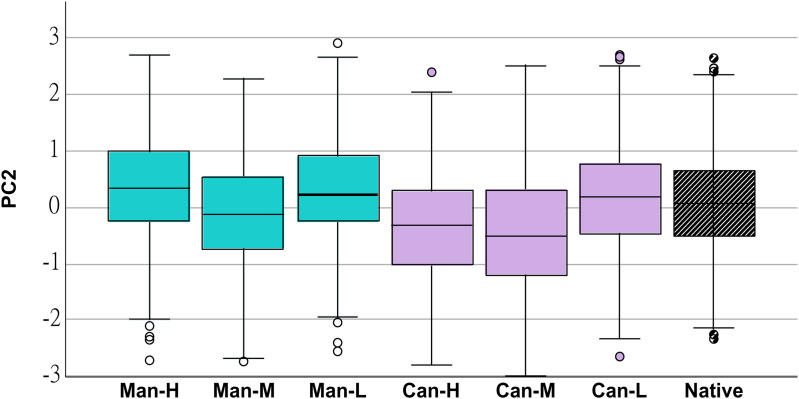
Summary of PC2 scores by accent strength. PC2 scores across accent strength conditions.

**Fig 4:**
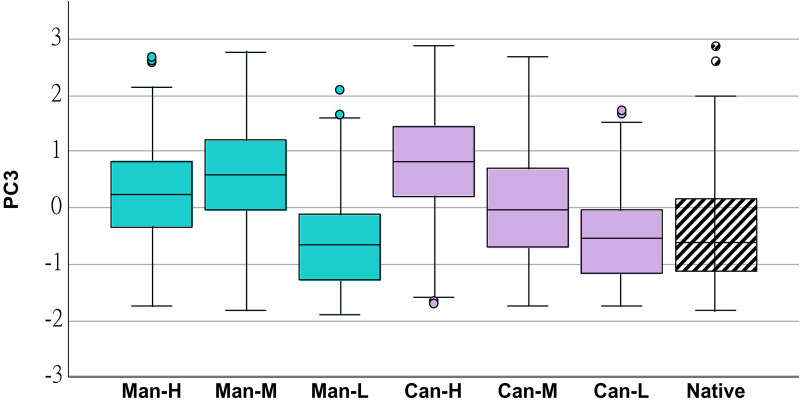
Summary of PC3 scores by accent strength. PC4 scores across accent strength conditions.

Figs 1–4 summarize the distribution of PCA-derived component scores across accent conditions. We have also included the summaries of mixed effects models and post-hoc comparisons of accent strength levels (*lmer()* for PC1 – PC4, *clmm()* for subsequent individual traits) in Supporting Information 5.

### Accentedness-related scores

Fig 1 shows PC4 scores (comprising Comprehensibility and Accentedness) across accent strength conditions. As shown in Fig 1, native English speech consistently yields higher PC4 scores than both Mandarin- and Cantonese-accented speech, indicating lower perceived accentedness and greater ease of understanding. A clear gradient is also visible within Chinese-accented speech, with weakly accented excerpts receiving higher scores than strongly accented ones, and Mandarin-accented speech generally outperforming Cantonese-accented speech across accent strength conditions.

Linear mixed effects model revealed that the fixed effect of Talker (in turn accent strength) was statistically significant, X2(13) = 1,087.1, p < .0001. Post-hoc pairwise comparisons showed that the native talker conditions yielded significantly higher PC4 scores (p < .0001) than the Chinese talker conditions, confirming that the native English samples were judged as less accented and more comprehensible. Similarly, the Mandarin-accented talkers yielded significantly higher (p < .0001) PC4 scores than the Cantonese-accented ones. For both Mandarin-accented and Cantonese-accented samples, the strongly accented conditions (H) yielded significantly lower (both p < .0001) PC4 scores than weakly accented conditions (L), suggesting that the former is more accented and less comprehensible. PC4 scores were significantly lower for the moderately Mandarin-accented samples than the strongly Mandarin-accented (p < .0001) and weakly Mandarin-accented (p < .0001) samples. No additional significant main effects were observed.

Supporting Information 4 summarizes the individual constituent scores. For comprehensibility, likelihood ratio tests likewise revealed a pronounced effect of talker (χ²(13) = 822.65, *p* < .001), and the inclusion of a subject-specific random slope for talker significantly enhanced model performance (χ²(104) = 192.62, *p* < .001). As expected, the comprehensibility ratings for native English speech were significantly higher than those for both Cantonese and Mandarin speakers (Cantonese: *p* < .0001, Mandarin: *p* < .0001) for all accent strength conditions, even for the weakly accented speech (Mandarin: *p* < .0001, Cantonese: *p* = .0001). Within Chinese-accented speech, Mandarin-accented short passages received higher comprehensibility ratings than Cantonese-accented ones (*p* < .0001). Strongly accented speech was significantly harder to understand than weakly accented speech (Mandarin: *p* = .0043, Cantonese: *p* < .0001).

For accentedness ratings, model comparisons indicated a robust main effect of talker (χ²(13) = 626.19, *p* < .001), confirming systematic differences in perceived accentedness across accent varieties. In detail, the accentedness ratings of native English short passages were significantly lower than those of both Cantonese- and Mandarin-accented ones (Cantonese: *p* < .0001, Mandarin: *p* < .0001). For both Mandarin- and Cantonese-accented passages, the strongly accented samples received significantly higher accentedness ratings than the weakly accented samples (*p* < .0001). Mandarin-accented passages were considered significantly less accented than Cantonese-accented ones (*p* < .0001).

It is noteworthy that neither the effect of the participants’ L1 nor the order of acquisition of languages was found to be significant for any of the three accent perception scores. No significant effects of L1 or acquisition order were observed; however, given the imbalance in subgroup sizes, this null result should be interpreted with caution.

### Language Attitudes

Below we present the responses to 18 attitudinal traits (6 each for superiority, attractiveness and dynamism) rated by the Cantonese and Mandarin speakers (see S4 File for details). Overall, native English speech was generally considered as superior to, more attractive, and more dynamic than both Cantonese- and Mandarin-accented speech. Mandarin-accented speech was rated more favorably than Cantonese-accented for most of the traits. Within Chinese-accented speech, weakly Cantonese-accented short passages were seen as generally superior to, more attractive and more dynamic than strongly and moderately accented ones. Weakly Mandarin-accented speech was rated lower for superiority and dynamism than strongly accented ones. Conversely, the attractiveness of strongly Mandarin-accented speech was higher than that of weakly accented speech.

### Superiority

Fig 2 shows PC1 scores (comprising predominantly superiority ratings, see S3 File) across accent strength conditions. Fig 2 illustrates that native English speech occupies the highest range of PC1 scores, followed by Mandarin-accented and then Cantonese-accented speech. Within each accent group, weakly accented samples tend to receive higher superiority ratings than strongly accented ones, indicating a consistent effect of accent strength on perceived status-related traits. Linear mixed effects model revealed that the fixed effect of Talker was statistically significant, *X*^2^(13) = 1,337.5, *p* < .0001. Post-hoc pairwise comparisons showed that the Native talker conditions yielded significantly higher PC1 scores (*p* < .0001) than the Chinese talker conditions, suggesting that the native English samples were judged as superior in terms of wealth, experience, competence, intelligence, and level of education, among other things. In contrast, the Mandarin-accented talkers yielded significantly lower (*p* < .0001) PC1 scores than the Cantonese-accented ones, indicating that the former was rated as less superior. For both Mandarin-accented and Cantonese-accented samples, the strongly accented conditions (H) yielded significantly lower (both *p* < .0001) PC1 scores than weakly accented conditions (L), suggesting that strongly Chinese-accented English is viewed as inferior to weakly Chinese-accented English.

Supporting Information 4 agrees with the PCA test that both the native English variant and the weakly Mandarin-accented variant consistently led in terms of perceived social ***superiority***. However, the superiority ratings on native English and weakly Mandarin-accented short passages were not significantly different for most traits except ‘rich’ (*p* < .0001). Ratings on Mandarin-accented excerpts were significantly higher than Cantonese-accented ones in all traits except for ‘rich’ and ‘experienced’. For ‘blue-collar’, ratings on native English speech were not significantly different from weakly Cantonese-accented speech (*p* = .6215).

### Attractiveness

As for ***attractiveness***, Fig 3 shows PC2 scores (comprising predominantly superiority ratings, see S3 File) across accent strength conditions. As shown in Fig 3, Mandarin-accented speech consistently receives the highest attractiveness ratings across conditions, exceeding both native English and Cantonese-accented speech. In contrast, strongly Cantonese-accented speech tends to occupy the lowest range of scores, while variation across accent strength appears less uniform for Mandarin-accented speech. A linear mixed effects model revealed that the fixed effect of Talker was statistically significant, *X*^2^(13) = 315.97, *p* < .0001. Post-hoc pairwise comparisons showed that the Native talker conditions yielded significantly higher PC2 scores (*p* < .0001) than the Cantonese talker conditions, suggesting that the native English samples were judged as more attractive (trustworthy, considerate, approachable, sincere, and friendly, among other things). Interestingly, both weakly (*p* = .0012) and strongly (*p* = .0001) Mandarin-accented talkers yielded significantly higher PC2 scores than the native talkers, indicating that the former were deemed more attractive. Strongly Cantonese-accented conditions yielded significantly lower (both *p* < .0001) PC2 scores than weakly accented conditions (L). In general, Mandarin-accented English yielded significantly higher PC2 scores than Cantonese-accented English (*p* < .0001).

### Dynamism

For ***dynamism***, Fig 4 shows PC3 scores (comprising predominantly dynamism ratings, see S3 File) across accent strength conditions. Fig 4 shows a different pattern from the previous dimensions, with Chinese-accented speech generally receiving higher dynamism ratings than native English. However, the distribution across accent groups is less systematic, with substantial overlap between Mandarin- and Cantonese-accented speech, suggesting weaker differentiation along this dimension. Linear mixed effects model revealed that the fixed effect of Talker was statistically significant, *X*^2^(13) = 864.86, *p* < .0001. Post-hoc pairwise comparisons showed that the Native talker conditions yielded significantly lower PC3 scores (*p* < .0001) than the Chinese talker conditions, suggesting that the native English samples were judged as less dynamic (i.e., *more* shy and passive). Mandarin-accented talkers and Cantonese-accented talkers were not rated as significantly different in terms of dynamism (*p* > .05). For both varieties, strongly accented talkers yielded significantly higher PC3 scores (i.e., more dynamic) than weakly accented ones (*p* < .0001).

Results in the Supporting Information 4 differed from the PCA analyses in that the ratings of native English variant consistently exceeded Cantonese- and Mandarin-accented ones except for the contrast of ‘aggressiveness’, in which the native English accent was rated as the most aggressive variant, a more negative rating than both Cantonese and Mandarin. Four of the six contrasts suggest a higher rating in Mandarin than in Cantonese (except for ‘shy’: *p* = .0002, ‘confident’: *p* = .6500). This result is in line with the PCA analyses that no consistent trend of perceptual bias can be found between Mandarin and Cantonese speech.

## Discussion

According to accent-related rating results, Chinese listeners did not find English spoken by Chinese L2 learners of English to be more easily understood than the native English variant. The native variant was favored over L2 ones in terms of both comprehensibility and accentedness, echoing its high prestige among both Cantonese- and Mandarin-speaking learners of English. As for Mandarin-Cantonese comparisons, surprisingly, PC 4 results show that both Cantonese and Mandarin speakers favored the Mandarin-accented excerpts over the Cantonese-accented ones. Weakly Cantonese-accented speech was regarded as significantly more challenging to understand than weakly Mandarin-accented speech, with the latter yielding accentedness ratings comparable to those of native English speech. These findings suggest that the asymmetric effects of accent strength across Mandarin and Cantonese suggest that ‘weak accent’ does not universally index desirability, but interacts with the sociopolitical meanings of the L1 from which the accent originates.

The consistently lower comprehensibility ratings as well as lower attitudinal trait scores on strongly accented learner speech may imply that the L2 speakers who sounded like native speakers of English were favored instead of being considered sell-outs or betrayers by the listeners. This differs from previous studies such as [36], which reported that in-group members who try to adopt a standard variety might risk being marginalized as a “sell-out” by their speech community. Importantly, comprehensibility ratings reflect perceived processing ease, whereas attitudinal traits index social evaluation. While these dimensions may be correlated in some contexts, they represent analytically distinct constructs and should not be interpreted as interchangeable.

The PCA results further indicate that comprehensibility and accentedness hold a shared perceptual basis. This aligns with previous research showing that while accentedness and comprehensibility are theoretically distinct, they are often strongly correlated in listener judgments. In the present data, this overlap suggests that listeners’ perception of accent strength is closely linked to perceived ease of understanding, rather than functioning as entirely independent evaluative dimensions.

Mandarin-accented passages were collectively rated as significantly less accented than Cantonese-accented ones. Such a marked difference may have a bearing on the phonological distance between English and Mandarin on the one hand, and English and Cantonese on the other. One plausible explanation for the observed differences is that Mandarin- and Cantonese-accented English exhibit distinct segmental and prosodic features, which may differentially affect listeners’ perceptual judgments. [37] These differences likely arise from specific phonetic patterns that are more or less salient to listeners in this context.

A similar listener bias was observed in the language attitude data. All language attitude rating patterns are shared by both Mandarin and Cantonese listeners. This echoes the fact that the effect of listeners’ L1 was non-significant in all the models fitted. The ratings of superiority yielded by Cantonese listeners on Cantonese are again lower than those from Mandarin listeners. The ratings of attractiveness stood similarly high for both Cantonese and Mandarin listeners. The effect of the order of acquisition within Cantonese speakers did not show significant differences in all three ratings either. Also, the absence of acquisition-order effects should be interpreted cautiously given the imbalance in subgroup sizes.

Work by McKenzie and colleagues has shown that learners may rate their own variety of English more positively and evaluate it in terms of solidarity and in-group identity, even when it is not associated with higher status or competence [26,38]. Such preferences therefore reflect identity play a significant role in accent perception. In contrast, the present results indicate that Mainland Cantonese-speaking learners do not consistently extend this in-group preference to status-related evaluations: Cantonese-accented English is rated lower on status and competence dimensions, despite its association with familiarity. This pattern aligns with previous findings that Chinese learners of English may internalize hierarchical language ideologies privileging standard or native varieties in academic and professional contexts [27,28]. However, Cantonese-accented speech was regarded as quite attractive despite low superiority. This pattern underscores that familiarity does not uniformly translate into positive status judgments and suggests that attractiveness and competence are independently negotiated in listeners’ accent evaluations. Previous research suggests that listeners evaluate L2 speech along a continuum between status-oriented and solidarity-oriented norms [36], for instance, in the extent to which familiar accents promote in-group affiliation. When a regional language comes under pressure from a more prestigious variety, evaluations may reflect either high status alongside high solidarity, or lower status coupled with higher solidarity, depending on speakers’ ideological orientations [39]. The present results suggest that dynamism ratings in this dataset may reflect perceived expressiveness or social energy of the speech rather than institutional authority or interpersonal closeness *per se*. However, given the weaker internal coherence of this dimension, these interpretations should be treated with caution. The overall contrast of strong vs. weak accent strengths within Mandarin speech preferred the strongly accented variant in terms of ‘friendliness’ over the weakly accented one as well as the native English variant. This agrees with previous literature showing that L2 listeners are confident with identifying with L2-accented speech in terms of solidarity. Conversely, Cantonese weak-accent speech was preferred, with a less marked advantage over the native English variant, suggesting that such confidence is not present. Since both Cantonese and Mandarin speakers rated Mandarin-accented English as more trustworthy, sincere and friendly than Cantonese-accented English, we may conclude that the solidarity aspect of the Cantonese accent is currently suffering from a decrease as opposed to its Mandarin counterpart. The trilingual participants’ L1-L2 acquisition order, namely Cantonese speakers with L1 Mandarin, L2 Cantonese and L3 English, and those with L1 Cantonese L2 Mandarin and L3 English, did not vary and remained non-significant in all accentedness and language attitude comparisons. This echoes our observation from other contrasts that Chinese speakers’ language attitude is relatively stable, potentially reflecting widely shared sociolinguistic hierarchies, and free from the effect of regional language variation. In addition, rather than reflecting language-specific familiarity, attitudes toward English accents appear to be shaped by broader, socially circulating ideologies about prestige and dynamism, which are similarly accessible to both Cantonese and Mandarin speakers. As a result, listeners from both groups converge in favoring Mandarin-accented English despite their differing linguistic backgrounds.

A limitation of the present study concerns the interpretability of the dynamism dimension. Unlike accentedness-related measures, which showed strong internal convergence across individual ratings and model-based analyses, the ratings underlying dynamism in Supporting Information 4 and the corresponding PCA-derived dynamism factor (PC3) exhibited weaker structural coherence, indicating that individual trait ratings did not consistently pattern by accent category. This dispersion suggests that dynamism-related judgments may be more sensitive to culturally specific interpretations of individual traits than to accent category per se. Accordingly, results concerning dynamism should be interpreted as exploratory tendencies rather than as stable accent effects, and future work with refined trait selection and targeted modeling is needed to establish the robustness of this dimension. We would like to point out that the classification of ‘Mandarin-only’ participants should be interpreted as reflecting dominant language use rather than categorical absence of exposure. Also, as participants were not explicitly asked to identify or label the accent varieties, the interpretation that the preferred L2 variant was perceived as Mandarin-accented is based on the controlled stimulus design and expert categorization rather than on participants’ metalinguistic awareness. This link between accent preference and Putonghua’s national symbolism should therefore be interpreted cautiously as an inferred association rather than a directly elicited one.

With regard to gender, no robust or systematic gender-based differences were observed in the present data; however, gender was not a primary analytical focus of this study. Previous research in the GBA has shown that evaluations of Cantonese and Putonghua may pattern differently along status and solidarity dimensions across social groups, including gender (e.g., [40]), and future research with targeted designs is needed to examine whether similar sociolinguistic stratification emerges in accent attitudes toward English varieties.

## Conclusion

In conclusion, Cantonese-accented English was perceived as less ***superior*** to and less ***dynamic*** than English- and Mandarin-accented English. The ratings on ***attractiveness*** were more varied, but the advantage of the Cantonese speech was meager. The results do not support our initial hypothesis that Cantonese L2 speakers would show preference towards Cantonese-accented speech among other variants – quite the opposite was found: both Cantonese and Mandarin speakers of English consider native English as the superior variant. The results also differ from the linguistic ecology described in Hong Kong previously, as in [4,5,11], who found that English represents status and Cantonese represents friendliness and solidarity. Mainland Cantonese participants yielded a distinct pattern, where they regarded another L2 variant of English (Mandarin) as the superior and more attractive one as opposed to their own. This preference may reflect the sociolinguistic prominence and perceived prestige of Putonghua in Mainland China. However, as accent identification and ideological orientation were not directly measured, this interpretation remains tentative. The findings agree with the prediction of [9] and [10] that regional languages (even powerful ones such as Cantonese) in China are losing their status to Putonghua as the standardization of which continues to proceed and its instrumental importance continues to strengthen.

We have seen that the linguistic power of Cantonese has been affected by the status of English and Mandarin, as shown by the language attitude ratings among Cantonese speakers in the current study. The shift of language power suggests that, in addition to advocating the use of the standard language, these findings also highlight the importance of preserving the Cantonese regional language, especially when it still has the function of unifying Cantonese speakers within the Bay Area.

## Supporting information

S1 File English Translation of the language attitude evaluation sheet. The actual sheet participants received had the items/traits randomized.
(DOCX)

S2 File Likelihood ratio tests for 21 dependent variables. (DOCX)

S3 File Summary of exploratory factor analysis results (N = 164). Loadings < ±.4 are not displayed.
(DOCX)

S4 File Descriptives and model summary of language attitude tasks.  + −5% is marked as 〇，5–10% as +, 10–15% as ++, and over 15% as +++. The same increments also apply for the markings of -, -- and ---. Negative traits are marked with an asterisk. Significance codes: p < 0.001: ‘***’, 0.001 < p < 0.01: ‘**’, 0.01 < p < 0.05: ‘*’, p > 0.05: ‘N.S.’.
(DOCX)

S5 File Summaries of mixed effects models and post-hoc comparisons of accent strength levels. *lmer()* for PC1 – PC4, *clmm()* for subsequent individual traits. Sex is sum-coded as [0.5 (male), −0.5 (female)].
(DOCX)

## References

[1] Lai ML. Exploring Language Stereotypes in Post‑colonial Hong Kong through the Matched-guise Test. JAPC. 2007;17(2):225–44. doi: 10.1075/japc.17.2.05lai

[2] Bourdieu P. Language and Symbolic Power. Cambridge: Polity Press. 1991.

[3] Giles H, Bourhis RY, Taylor DM. Towards a theory of language in ethnic group relations. In: Giles H, editor. Language, Ethnicity and Intergroup Relations. New York: Academic Press. 1977. p. 307–48.

[4] Gibbons J. Code-mixing and code choice: A Hong Kong case study. Clevedon: Multilingual Matters. 1987.

[5] Lai M. Hong Kong students’ attitudes towards Cantonese, Putonghua and English after the change of sovereignty. J Multiling Multicult Dev. 2001;22:112–33. doi: 10.1080/01434630108666428

[6] Ladegaard HJ, Chan KLR. Teachers’ attitudes towards varieties of Hong Kong English. EWW. 2022;44(2):251–75. doi: 10.1075/eww.21060.lad

[7] Bolton K, Bacon-Shone J. The statistics of English across Greater China. World Englishes. 2020;39:495–512. doi: 10.1111/weng.12478

[8] Bai J. Language attitude and the spread of Standard Chinese in China. Lang Probl Lang Plann. 1994;18:128–38. doi: 10.1075/lplp.18.2.03bai

[9] Zhou M. The spread of Putonghua and language attitude changes. J Asia Pac Commun. 2001;11(2):231–53. doi: 10.1075/japc.11.2.07zho

[10] Ng DF, Zhao J. Cantonese speakers’ language attitudes in Mainland China. J Multiling Multicult Dev. 2015;36:357–71.

[11] Kalmar I, Yong Z, Hong X. Language attitudes in Guangzhou, China. Lang Soc. 1987;16:499–508. doi: 10.1017/S0047404500000348

[12] Fang X. A study on education in Mandarin in Cantonese-speaking areas. Appl Linguist. 2003;:39–42.

[13] Murphy S. Second language transfer during third language acquisition. Work Pap TESOL Appl Linguist. 2003;3. doi: 10.7916/salt.v3i1.1632

[14] Chen HC, Han QW. L3 phonology: Contributions of L1 and L2 to L3 pronunciation learning by Hong Kong speakers. Int J Multiling. 2019;16:492–512.

[15] Han Q, Tian J, Chen H. L3 prosody: Cross-linguistic influence of prosodic features in Mandarin and English by Cantonese multilinguals. In: Megan M, Flynn S, Fernández-Berkes E, editors. L3 Development After the Initial State. Amsterdam: John Benjamins. 2023. p. 96–121. doi: 10.1075/sibil.65.05han

[16] Wang D, Nance C. Third language phonological acquisition. Lang Linguist Compass. 2023;2023:e12497. doi: 10.1111/lnc3.12497

[17] Munro MJ, Derwing TM. Evaluations of foreign accent. Lang Test. 1994;11:253–66.

[18] Garrett P. Attitudes to Language. Cambridge: Cambridge University Press. 2010.

[19] Kircher R, Zipp L. Research Methods in Language Attitudes. Cambridge: Cambridge University Press. 2022.

[20] Munro MJ. A Primer on Accent Discrimination in the Canadian Context. TESL. 2003;20(2):38. doi: 10.18806/tesl.v20i2.947

[21] Edwards JH, Chan KLR, Lam T, Wang Q. Social factors and the teaching of pronunciation: What the research tells us. RELC J. 2021;52:35–47.

[22] Cargile AC, Giles H, Ryan EB, Bradac JJ. Language attitudes as a social process: A conceptual model and new directions. Lang Commun. 1994;14:211–36. doi: 10.1016/0271-5309(94)90001-9

[23] Zahn CJ, Hopper R. Measuring language attitudes. J Lang Soc Psychol. 1985;4:113–23.

[24] Schoel C, Roessel J, Eck J, Janssen J, Petrovic B, Rothe A. Attitudes towards languages (AToL) scale. J Lang Soc Psychol. 2012;32:21–45. doi: 10.1177/0261927X12457922

[25] McKenzie RM, Gilmore A. Japanese university students’ evaluations of English language diversity. Int J Appl Linguist. 2017;27:152–75. doi: 10.1111/ijal.12110

[26] Author A, Author B, Author C. Accent stereotyping among Mandarin Chinese learners of English in academic contexts. 2022.

[27] Author A, Author B, Author C. Prejudice Against L2-Accented English in Career Settings: A Study of Chinese Learners. 2023.

[28] Squizzero R. The Effects of Perceived Ethnicity and Prosodic Accuracy on Intelligibility, Comprehensibility, and Accentedness in L2 Mandarin Chinese. Lang Speech. 2025;:238309251361010. doi: 10.1177/00238309251361010 40956078

[29] Weinberger S. Speech Accent Archive. George Mason University. https://accent.gmu.edu/. 2015.

[30] Munro MJ, Derwing TM. Foreign accent, comprehensibility, and intelligibility. Lang Learn. 1995;45:73–97. doi: 10.1111/j.1467-1770.1995.tb00963.x

[31] Kang O, Rubin D, Pickering L. Suprasegmental measures of accentedness and judgments of English learner proficiency. Mod Lang J. 2010;94:554–66. doi: 10.1111/j.1540-4781.2010.01091.x

[32] Setter J, Wong CSP, Chan BHS. Hong Kong English. In: Schreier D, editor. The Lesser-Known Varieties of English. Cambridge: Cambridge University Press. 2010. p. 53–69.

[33] Osgood CE, May WH, Miron MS. Cross-cultural universals of affective meaning. Urbana, IL: University of Illinois Press. 1975.

[34] Kuznetsova A, Brockhoff PB, Christensen RHB. lmerTest package: Tests in linear mixed effects models. J Stat Softw. 2017;82:1–26. doi: 10.18637/jss.v082.i13

[35] Christensen RHB, Brockhoff PB. Analysis of sensory ratings data with cumulative link models. J Soc Fr Stat. 2013;154:58–79.

[36] Giles H, Edwards JR. Attitudes to language: Past, present, and future. In: Malmkjaer K, editor. The Routledge Linguistics Encyclopedia. 3rd ed. London: Routledge. 2010. p. 35–40.

[37] Matthews S, Yip V. Cantonese: A Comprehensive Grammar. London: Routledge. 1994.

[38] McKenzie RM. The Sociolinguistics of Variety Identification and Categorisation. Cambridge: Cambridge University Press; 2015.

[39] Ryan EB, Giles H, Sebastian RJ. An integrative perspective for the study of attitudes toward language variation. In: Ryan EB, Giles H, editors. Attitudes Towards Language Variation. London: Edward Arnold. 1982. p. 1–19.

[40] Wang X, Ladegaard HJ. Language attitudes and gender in China. Lang Aware. 2008;17:57–77. doi: 10.2167/la422.0

