# Supplementary material for: Regional language effects on accent perception and language attitude: The case of mandarin vs. cantonese speakers in mainland China
Source: PLoS One. 2026 Jul 6;21(7):e0352330. doi: 10.1371/journal.pone.0352330 (PMC13336171; doi:10.1371/journal.pone.0352330)
Supplement: S1 File — The actual sheet participants received had the items/traits randomized. (DOCX) [file pone.0352330.s001.docx]

**Supporting Information 1: English Translation of the language attitude evaluation sheet. The actual sheet participants received had the items/traits randomized.**

Directions: Listen to the 14 speakers and decide to what extent you agree that they match the following traits. Use your first intuition. 5 = strongly agree 1 = strongly disagree. Reversely coded traits were marked with an asterisk.

Cluster 1 Superiority

1.1. Intelligent

1.2. Educated

1.3. Competent

1.4. Rich

*1.5. Blue-collar

1.6. Experienced

Cluster 2 Attractiveness

2.1. Friendly

*2.2. Arrogant

2.3. Sincere

2.4. Approachable

2.5. Considerate

2.6. Trustworthy

Cluster 3 Dynamism

3.1 Industrious

*3.2. Aggressive

3.3. Trendy

*3.4. Passive

*3.5. Shy

3.6. Confident
