# Supplementary material for: Regional language effects on accent perception and language attitude: The case of mandarin vs. cantonese speakers in mainland China
Source: PLoS One. 2026 Jul 6;21(7):e0352330. doi: 10.1371/journal.pone.0352330 (PMC13336171; doi:10.1371/journal.pone.0352330)
Supplement: S2 File — (DOCX) [file pone.0352330.s002.docx]

**Supporting Information 2: Likelihood ratio tests for 20 dependent variables.**

|  |  |  | IV | npar | AIC | logLik | LR.stat | df | Pr(>Chisq) |
| --- | --- | --- | --- | --- | --- | --- | --- | --- | --- |
| Accent Perception | | Accentedness | Talker | 18 | 6,022 | -2,993 | 626.190 | 13 | < .001 |
|  |  |  | (Talker\|Subject) | 122 | 5,522 | -2,639 | 707.280 | 104 | < .001 |
|  |  | Comprehensibility | Talker | 18 | 5,273 | -2,619 | 822.650 | 13 | < .001 |
|  |  |  | (Talker\|Subject) | 122 | 5,289 | -2,522 | 192.620 | 104 | < .001 |
| Language attitude | Superiority | Intelligent | Talker | 18 | 5,033 | -2,499 | 882.730 | 13 | < .001 |
|  |  |  | (Talker\|Subject) | 122 | 5,089 | -2,423 | 152.290 | 104 | .001 |
|  |  | Educated | Talker | 18 | 5,018 | -2,491 | 1,127.900 | 13 | < .001 |
|  |  |  | (Talker\|Subject) | 122 | 5,030 | -2,393 | 195.840 | 104 | < .001 |
|  |  | Competent | Talker | 18 | 5,151 | -2,558 | 1,209.900 | 13 | < .001 |
|  |  | Rich | Talker | 18 | 5,081 | -2,523 | 813.500 | 13 | < .001 |
|  |  |  | (Talker\|Subject) | 122 | 5,073 | -2,414 | 216.700 | 104 | < .001 |
|  |  | * BlueCollar | Talker | 18 | 5,167 | -2,566 | 239.584 | 13 | < .001 |
|  |  |  | #YrsStudyEng | 19 | 5,165 | -2,564 | 3.987 | 1 | .046 |
|  |  | Experienced | Talker | 18 | 5,419 | -2,692 | 880.090 | 13 | < .001 |
|  |  |  | (Talker\|Subject) | 122 | 5,444 | -2,600 | 182.980 | 104 | < .001 |
|  | Attractiveness | Friendly | Talker | 18 | 5,334 | -2,649 | 176.800 | 13 | < .001 |
|  |  |  | (Talker\|Subject) | 122 | 5,367 | -2,562 | 174.100 | 104 | < .001 |
|  |  | * Arrogant | Talker | 18 | 4,822 | -2,393 | 574.630 | 13 | < .001 |
|  |  |  | (Talker\|Subject) | 122 | 4,861 | -2,309 | 168.590 | 104 | < .001 |
|  |  | Sincere | Talker | 18 | 5,405 | -2,685 | 144.546 | 13 | < .001 |
|  |  |  | AcqOrder | 23 | 5,402 | -2,678 | 13.152 | 5 | .022 |
|  |  |  | (Talker\|Subject) | 127 | 5,465 | -2,605 | 145.227 | 104 | .005 |
|  |  | Approachable | Talker | 18 | 5,546 | -2,755 | 189.066 | 13 | < .001 |
|  |  |  | AcqOrder | 23 | 5,539 | -2,746 | 17.232 | 5 | .004 |
|  |  |  | (Talker\|Subject) | 127 | 5,573 | -2,660 | 173.787 | 104 | < .001 |
|  |  | Considerate | Talker | 18 | 5,202 | -2,583 | 227.830 | 13 | < .001 |
|  |  |  | (Talker\|Subject) | 122 | 5,265 | -2,511 | 145.180 | 104 | .005 |
|  |  | Trustworthy | Talker | 18 | 5,429 | -2,697 | 434.330 | 13 | < .001 |
|  |  |  | (Talker\|Subject) | 122 | 5,446 | -2,601 | 191.010 | 104 | < .001 |
|  | Dynamism | Industrious | Talker | 18 | 5,312 | -2,638 | 455.430 | 13 | < .001 |
|  |  |  | (Talker\|Subject) | 122 | 5,292 | -2,524 | 227.380 | 104 | < .001 |
|  |  | * Aggressive | Talker | 18 | 4,610 | -2,287 | 735.701 | 13 | < .001 |
|  |  |  | Gender | 19 | 4,607 | -2,284 | 5.333 | 1 | .021 |
|  |  | Trendy | Talker | 18 | 5,260 | -2,612 | 851.093 | 13 | < .001 |
|  |  |  | Gender | 19 | 5,257 | -2,609 | 5.520 | 1 | .019 |
|  |  | * Passive | Talker | 18 | 5,516 | -2,740 | 794.400 | 13 | < .001 |
|  |  |  | (Talker\|Subject) | 122 | 5,533 | -2,645 | 190.570 | 104 | < .001 |
|  |  | * Shy | Talker | 18 | 5,447 | -2,705 | 919.250 | 13 | < .001 |
|  |  | Confident | Talker | 18 | 5,359 | -2,662 | 1,286.500 | 13 | < .001 |
|  |  |  | (Talker\|Subject) | 122 | 5,362 | -2,559 | 205.530 | 104 | < .001 |
