# Supplementary material for: Regional language effects on accent perception and language attitude: The case of mandarin vs. cantonese speakers in mainland China
Source: PLoS One. 2026 Jul 6;21(7):e0352330. doi: 10.1371/journal.pone.0352330 (PMC13336171; doi:10.1371/journal.pone.0352330)
Supplement: S3 File — Loadings < ±.4 are not displayed. (DOCX) [file pone.0352330.s003.docx]

**Supporting Information 3: Summary of exploratory factor analysis results (N = 164). Loadings < ±.4 are not displayed. Negative traits are marked with an asterisk.**

|  |  | **Pattern Matrix** | | | | **Structure Matrix** | | | |
| --- | --- | --- | --- | --- | --- | --- | --- | --- | --- |
|  |  | **Component** | | | | | | | |
|  |  | **1** | **2** | **3** | **4** | **1** | **2** | **3** | **4** |
| Dynamic | *** Aggressive** | .864 |  |  |  | .796 |  |  |  |
| Attract | *** Arrogant** | .834 |  |  |  | .765 |  |  |  |
| Dynamic | **Trendy** | .673 |  |  |  | .748 |  | -.419 |  |
| Superior | **Rich** | .602 |  |  |  | .726 |  | -.447 | .490 |
| Dynamic | **Confident** | .573 |  |  |  | .753 |  | -.650 | .466 |
| Superior | **Experienced** | .526 |  |  |  | .639 | .402 |  | .502 |
| Superior | **Competent** | .508 |  |  |  | .715 | .438 | -.565 | .642 |
| Superior | **Intelligent** | .472 |  |  |  | .653 | .458 | -.509 | .575 |
| Superior | **Educated** | .423 |  |  |  | .645 | .452 | -.539 | .670 |
| Attract | **Friendly** |  | .862 |  |  |  | .840 |  |  |
| Attract | **Approachable** |  | .827 |  |  |  | .767 |  |  |
| Attract | **Sincere** |  | .824 |  |  |  | .790 |  |  |
| Attract | **Considerate** |  | .748 |  |  |  | .756 |  |  |
| Attract | **Trustworthy** |  | .626 |  |  |  | .721 |  | .505 |
| Dynamic | **Industrious** |  | .569 |  |  | .420 | .651 |  | .459 |
| Superior | *** BlueCollar** |  |  | .824 |  |  |  | .738 |  |
| Dynamic | *** Passive** |  |  | .791 |  | -.440 |  | .831 |  |
| Dynamic | *** Shy** |  |  | .658 |  | -.562 |  | .759 |  |
| Accent | **Accentedness** |  |  |  | -.945 |  |  |  | -.831 |
| Accent | **Comprehensibility** |  |  |  | .718 |  |  |  | .779 |
|  | Extraction Method: Principal Component Analysis.  Rotation Method: Oblimin with Kaiser Normalization.  a. Rotation converged in 10 iterations. | | | | | | | | |
