# Supplementary material for: Regional language effects on accent perception and language attitude: The case of mandarin vs. cantonese speakers in mainland China
Source: PLoS One. 2026 Jul 6;21(7):e0352330. doi: 10.1371/journal.pone.0352330 (PMC13336171; doi:10.1371/journal.pone.0352330)
Supplement: S4 File — + −5% is marked as 〇，5–10% as +, 10–15% as ++, and over 15% as +++. The same increments also apply for the markings of -, -- and ---. Negative traits are marked with an asterisk. Significance codes: p < 0.001: ‘***’, 0.001 < p < 0.01: ‘**’, 0.01 < p < 0.05: ‘*’, p > 0.05: ‘N.S.’. (DOCX) [file pone.0352330.s004.docx]

**Supporting Information 4: Descriptives and model summary of language attitude tasks. +-5% is marked as 〇，5-10% as +, 10-15% as ++, and over 15% as +++. The same increments also apply for the markings of -, -- and ---. Negative traits are marked with an asterisk. Significance codes: p<0.001: ‘***’, 0.001<p<0.01: ‘**’, 0.01<p<0.05: ‘*’, p>0.05: ‘N.S.’.**

|  | **Task** | **Cantonese** | | | | **Mandarin** | | | | **English** | **Significance** | | |
| --- | --- | --- | --- | --- | --- | --- | --- | --- | --- | --- | --- | --- | --- |
|  |  | STR | MOD | WEK | AVG | STR | MOD | WEK | AVG |  | **Can-Man (sig)** | **Can-Eng (sig)** | **Man-Eng (sig)** |
| Superiority | Intelligent | --- | 〇 | +++ | 2.92 | - | +++ | +++ | 3.06 | 3.70 | *** | *** | *** |
|  | Educated | --- | 〇 | +++ | 3.05 | - | +++ | +++ | 3.24 | 3.99 | *** | *** | *** |
|  | Competent | --- | 〇 | +++ | 2.86 | - | +++ | +++ | 3.00 | 3.89 | *** | *** | *** |
|  | Rich | --- | 〇 | +++ | 2.71 | - | +++ | +++ | 2.71 | 3.53 | N.S | *** | *** |
|  | *Blue collar | +++ | 〇 | --- | 2.24 | 〇 | +++ | -- | 2.06 | 1.76 | ** | *** | *** |
|  | Experienced | --- | + | +++ | 2.76 | - | +++ | +++ | 2.78 | 3.57 | N.S | *** | *** |
| Attractiveness | Friendly | 〇 | - | + | 3.10 | 〇 | +++ | 〇 | 3.42 | 3.20 | *** | ** | *** |
|  | *Arrogant | --- | ++ | +++ | 2.03 | --- | +++ | +++ | 1.77 | 2.55 | *** | *** | *** |
|  | Sincere | 〇 | - | + | 3.01 | + | +++ | 〇 | 3.27 | 3.11 | *** | * | ** |
|  | Approachable | 〇 | - | + | 2.95 | + | +++ | 〇 | 3.21 | 2.86 | *** | N.S | *** |
|  | Considerate | - | - | ++ | 2.76 | 〇 | +++ | 〇 | 3.06 | 2.95 | *** | *** | N.S |
|  | Trustworthy | -- | 〇 | ++ | 2.90 | 〇 | +++ | ++ | 3.19 | 3.49 | *** | *** | *** |
| Dynamism | Industrious | --- | 〇 | ++ | 2.85 | 〇 | +++ | ++ | 3.13 | 3.42 | *** | *** | *** |
|  | *Aggressive | --- | +++ | +++ | 2.03 | --- | +++ | +++ | 1.76 | 2.64 | *** | *** | *** |
|  | Trendy | --- | - | +++ | 2.28 | - | +++ | +++ | 2.35 | 3.27 | N.S | *** | *** |
|  | *Passive | +++ | - | --- | 2.56 | + | +++ | --- | 2.55 | 1.83 | N.S | *** | *** |
|  | *Shy | +++ | -- | --- | 2.43 | ++ | +++ | --- | 2.61 | 1.84 | *** | *** | *** |
|  | Confident | --- | + | +++ | 2.98 | - | +++ | +++ | 2.96 | 3.86 | N.S | *** | *** |
