# Supplementary material for: Regional language effects on accent perception and language attitude: The case of mandarin vs. cantonese speakers in mainland China
Source: PLoS One. 2026 Jul 6;21(7):e0352330. doi: 10.1371/journal.pone.0352330 (PMC13336171; doi:10.1371/journal.pone.0352330)
Supplement: S5 File — Sex is sum-coded as [0.5 (male), −0.5 (female)]. (DOCX) [file pone.0352330.s005.docx]

**Supporting Information 5: Summaries of mixed effects models and post-hoc comparisons of accent strength levels. *lmer()* for PC1 – PC4, *clmm()* for subsequent individual traits. Sex is sum-coded as [0.5 (male), -0.5 (female)].**

| **PC1 ~ (1 \| ID) + Talker** |
| --- |
| 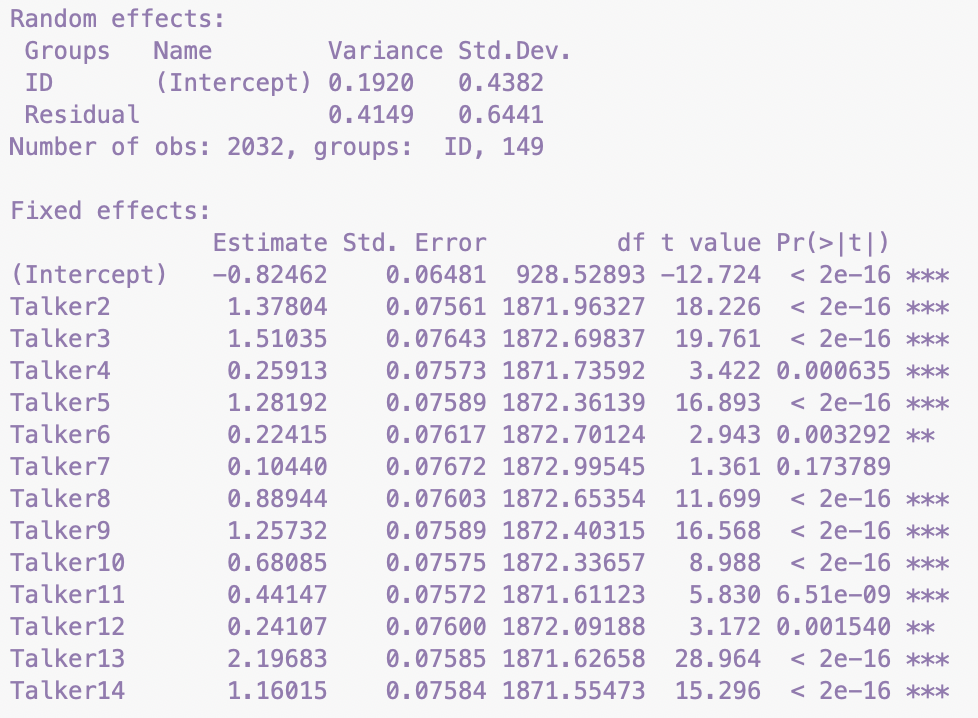  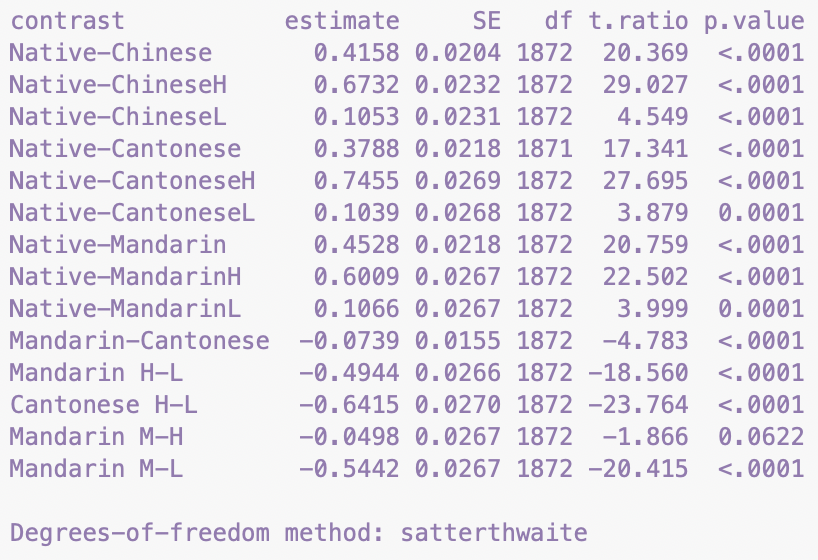 |

| **PC2 ~ (1 \| ID) + Talker** |
| --- |
| 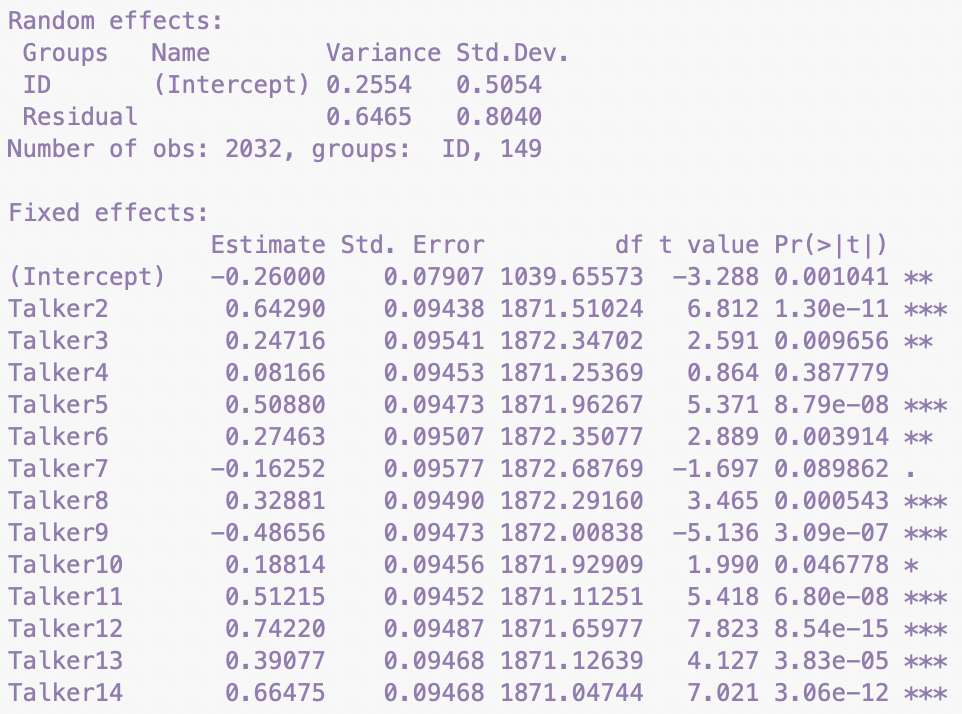  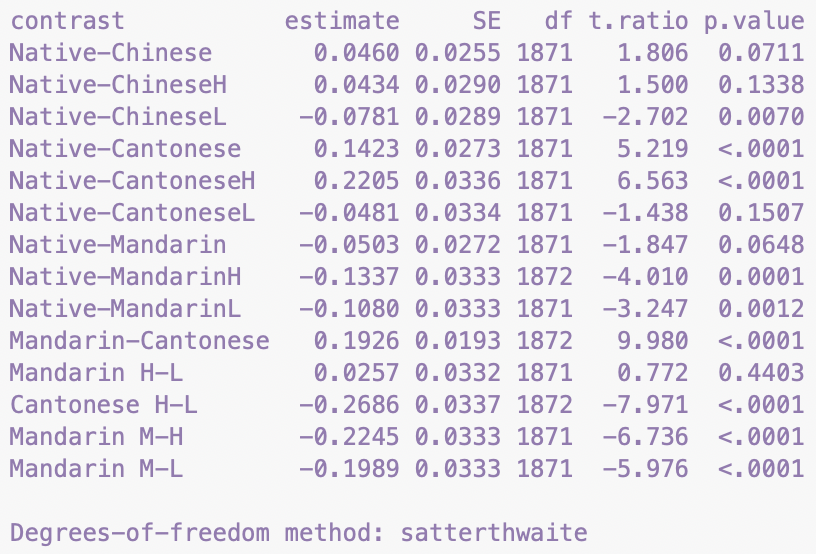 |

| **PC3 ~ (1 \| ID) + Talker** |
| --- |
| 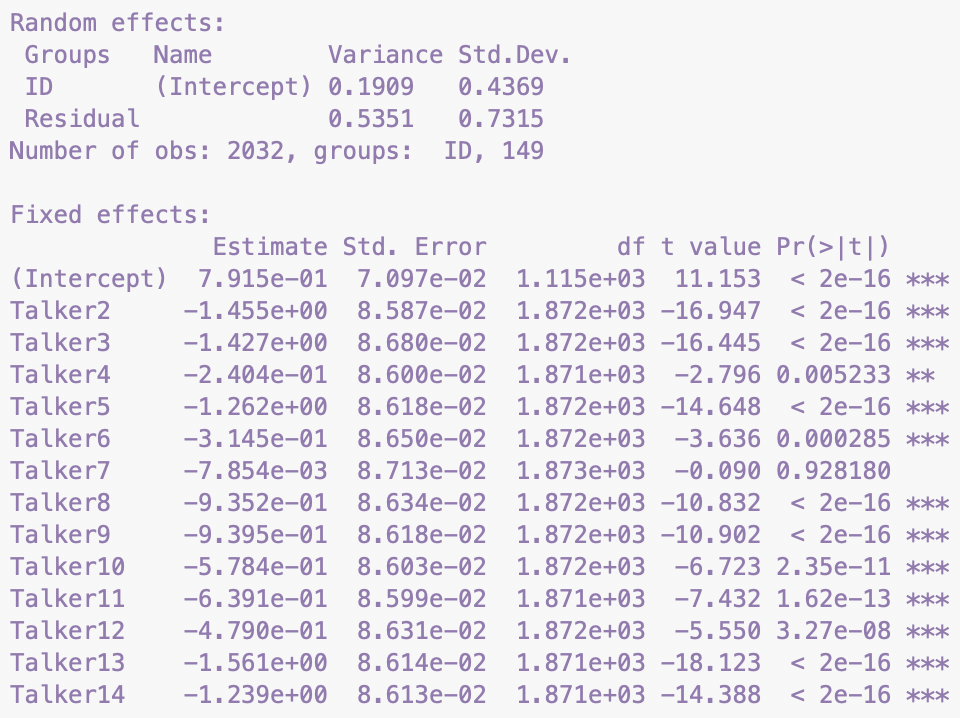  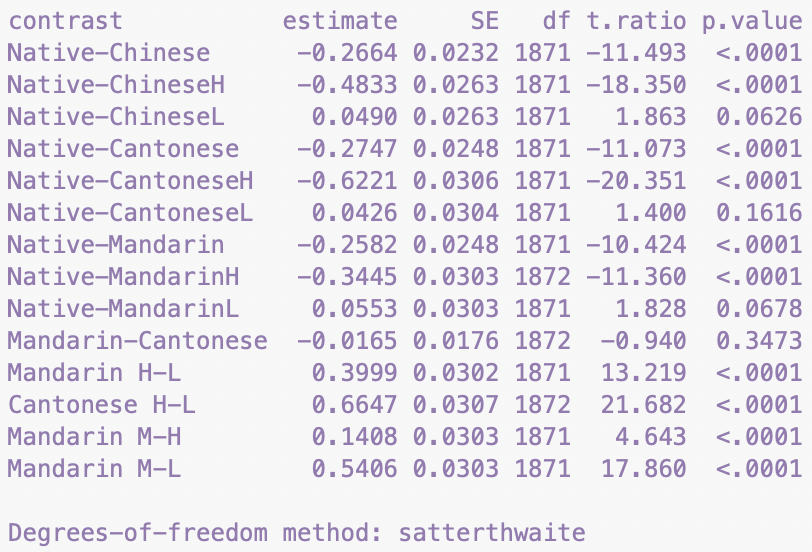 |

| **PC4 ~ (1 \| ID) + Talker** |
| --- |
| 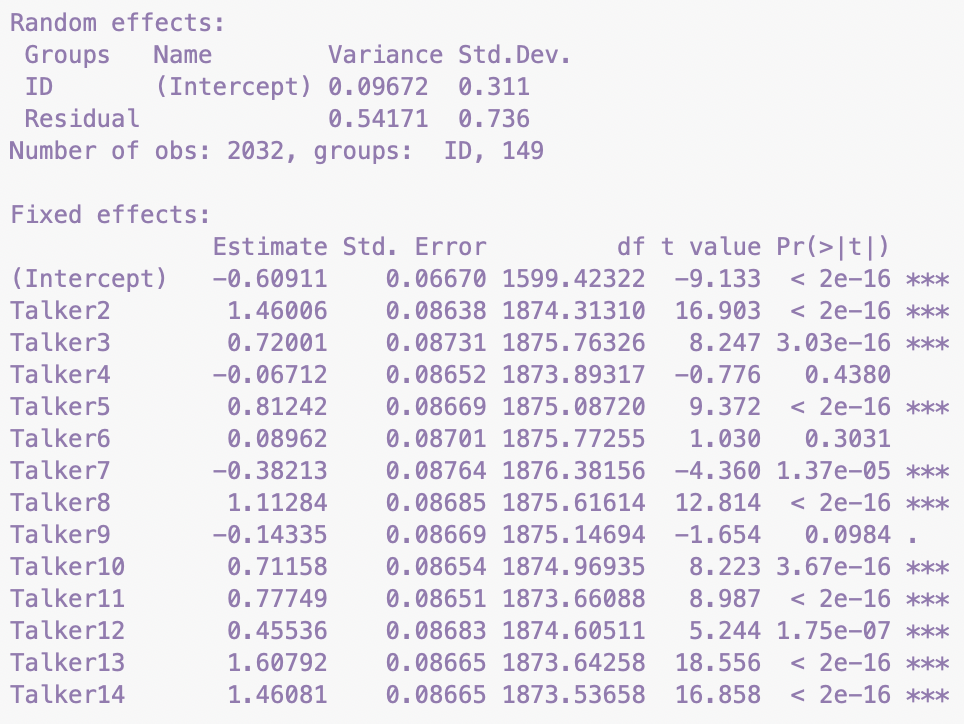  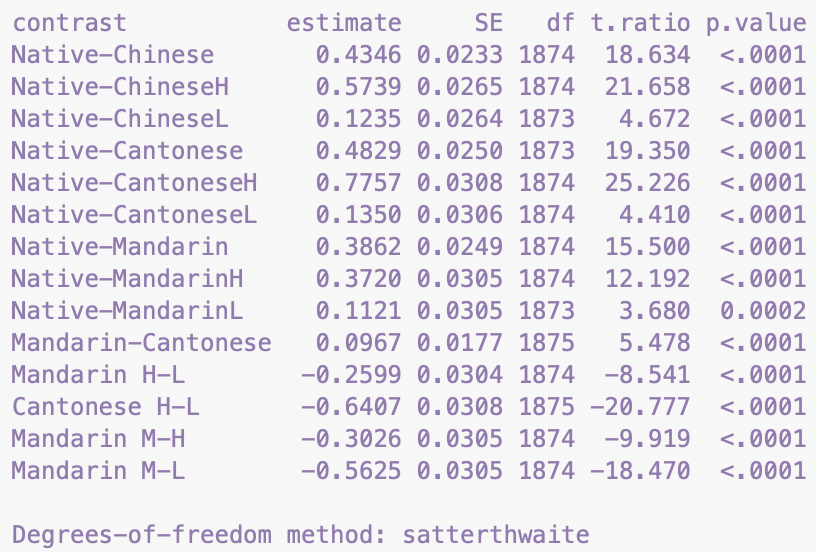 |

| **Accentedness ~ Talker + (Talker \| ID)** |
| --- |
| 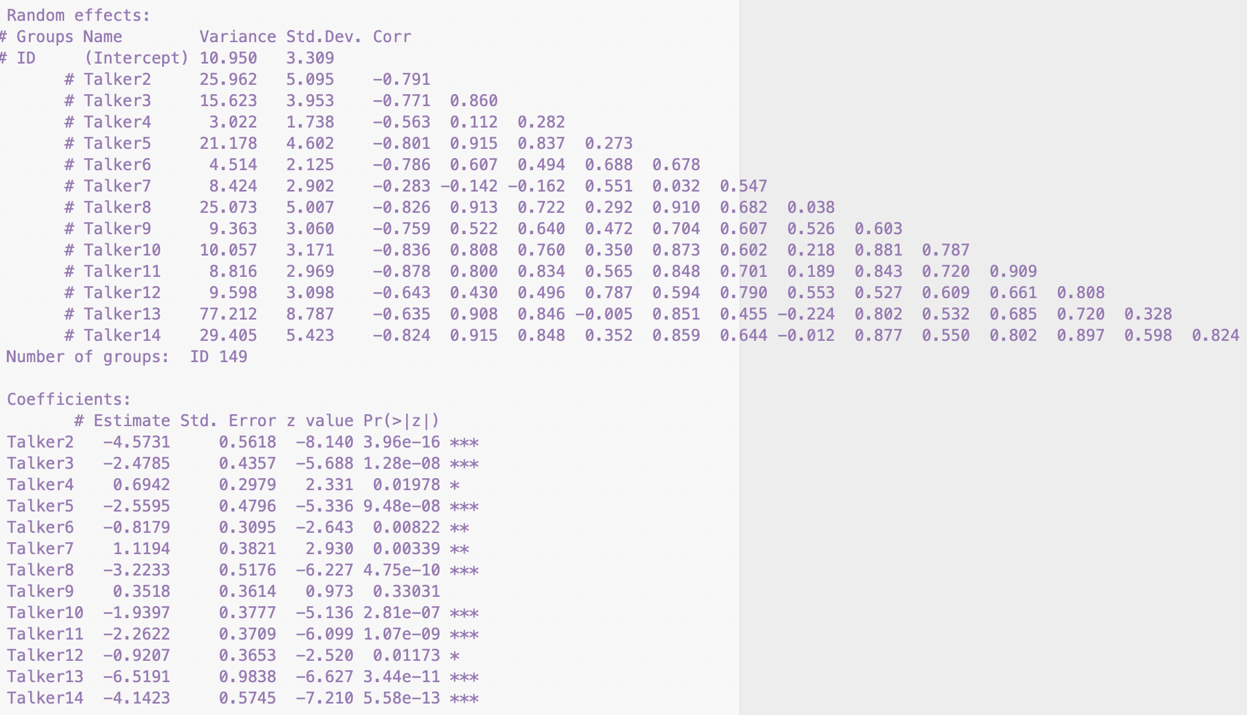  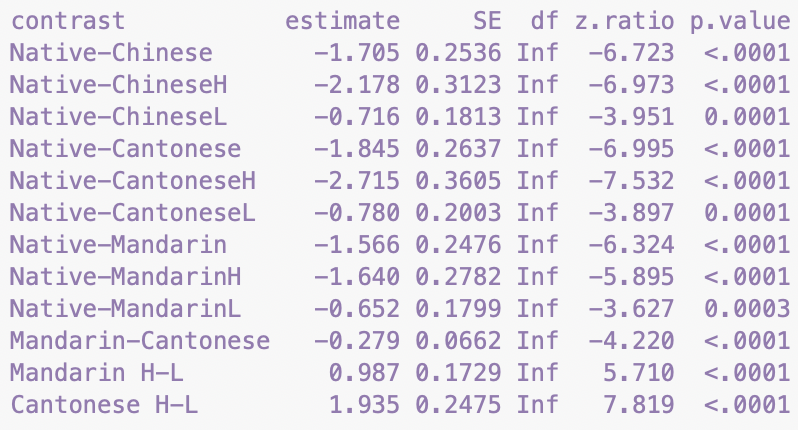 |

| **Comprehensibility ~ Talker + (Talker \| ID)** |
| --- |
| 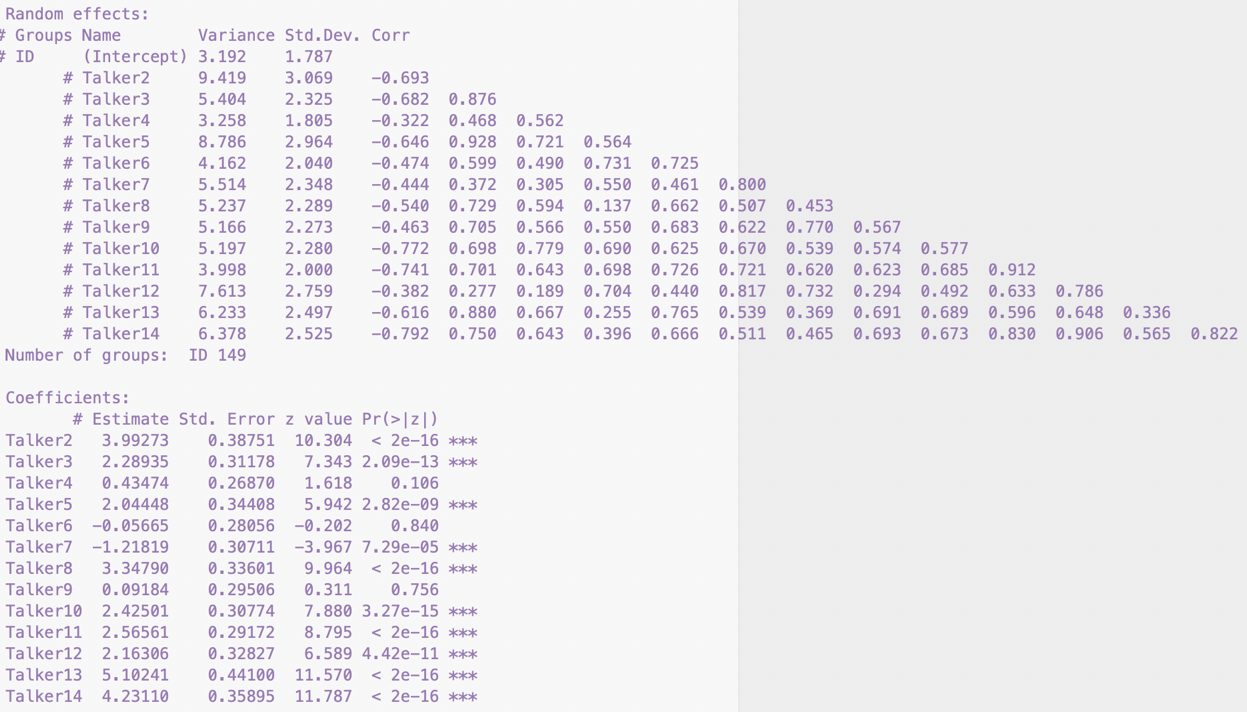  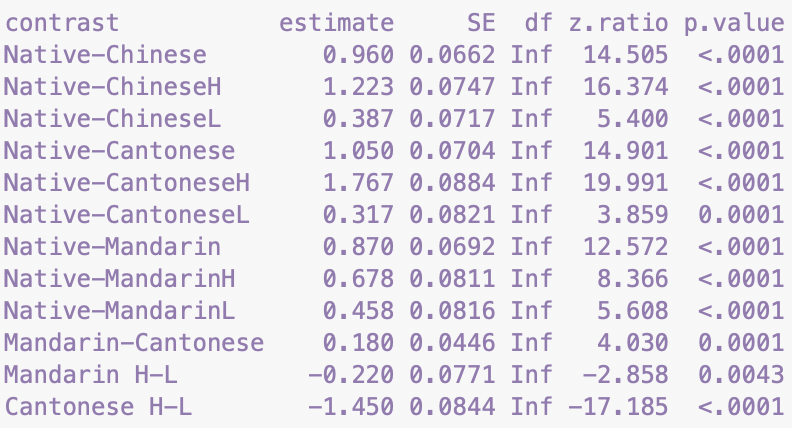 |

| **Intelligent ~ Talker + (Talker \| ID)** |
| --- |
| 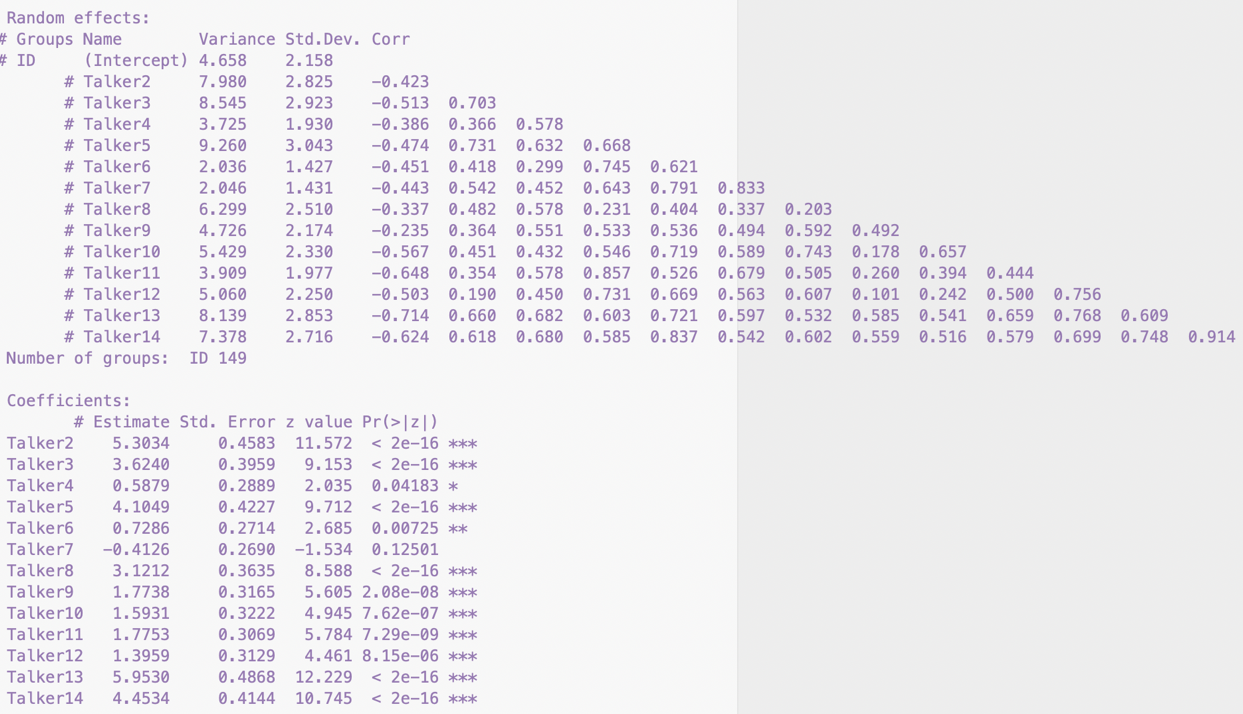  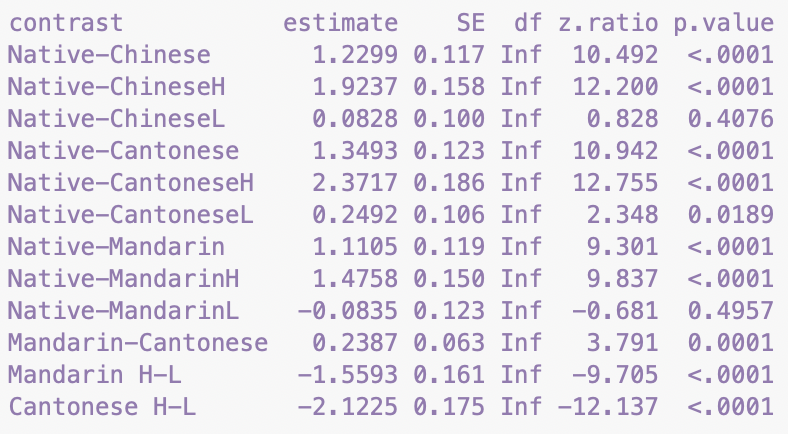 |

| **Educated ~ Talker + (Talker \| ID)** |
| --- |
| 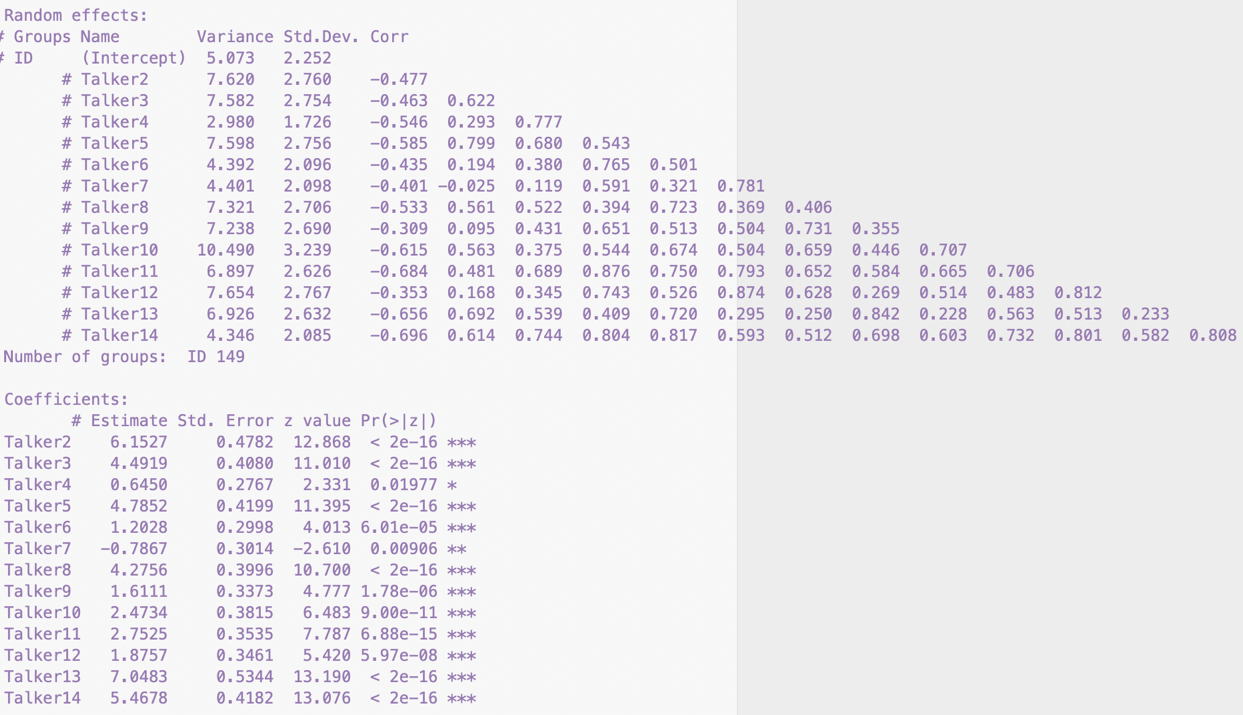  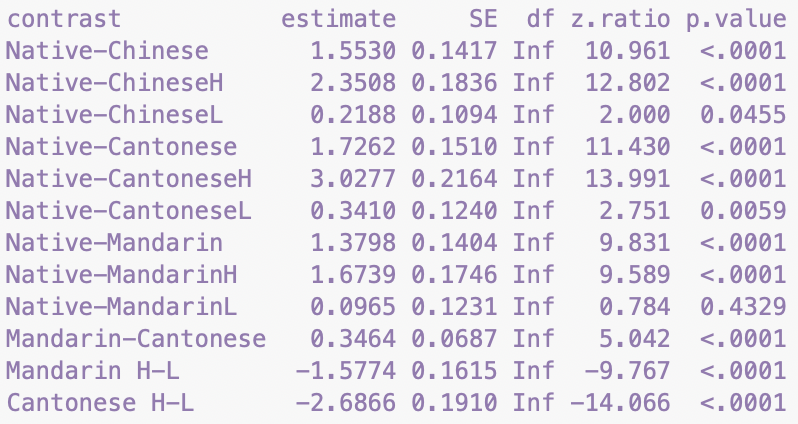 |

| **Competent ~ Talker + (1 \| ID)** |
| --- |
| 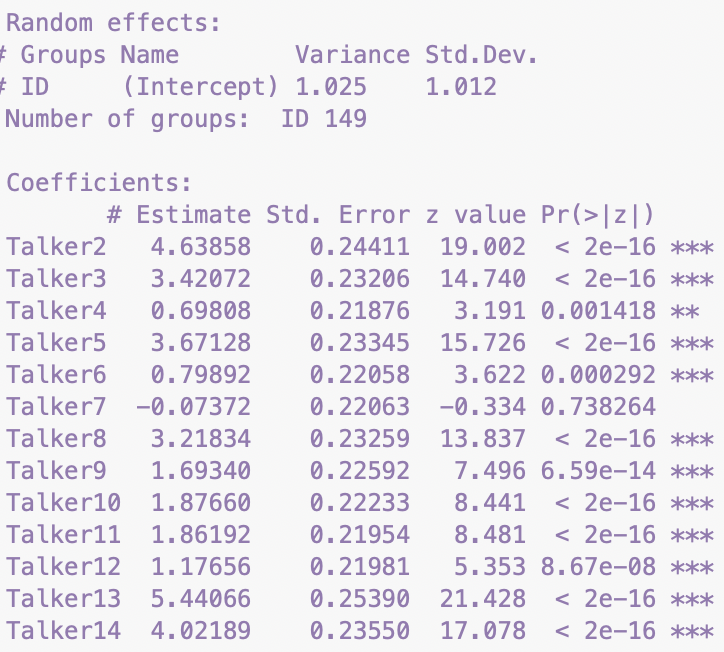  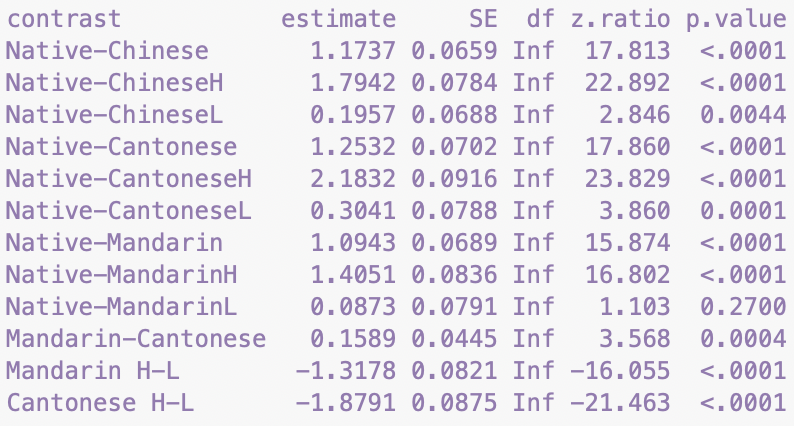 |

| **Rich ~ Talker + (Talker \| ID)** |
| --- |
| 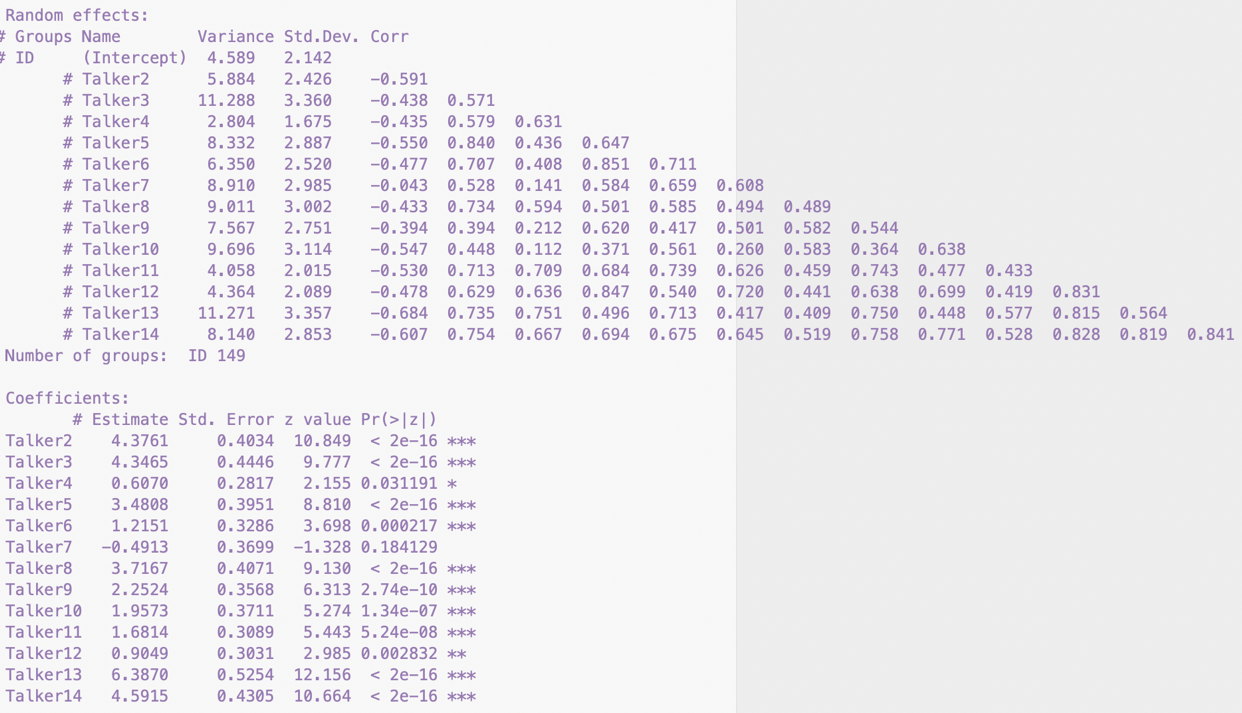  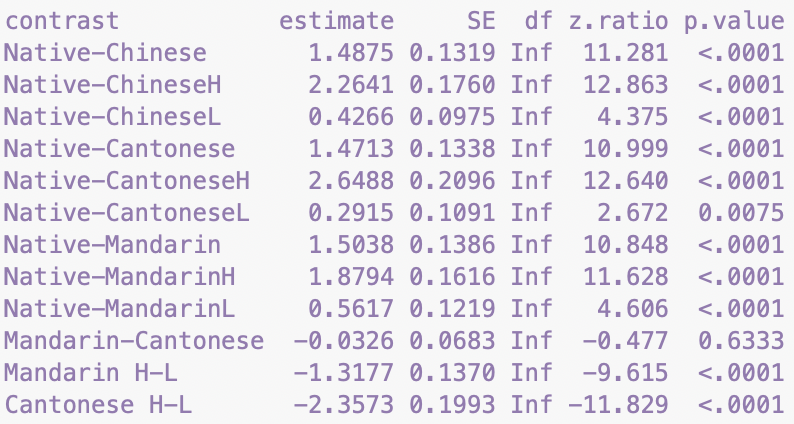 |

| *** BlueCollar ~ (1 \| ID) + Talker + EngYrs** |
| --- |
| 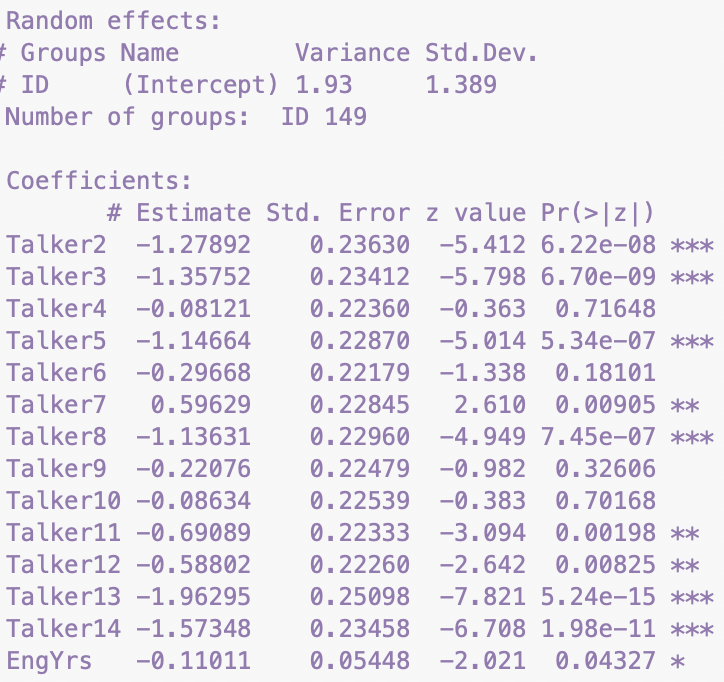  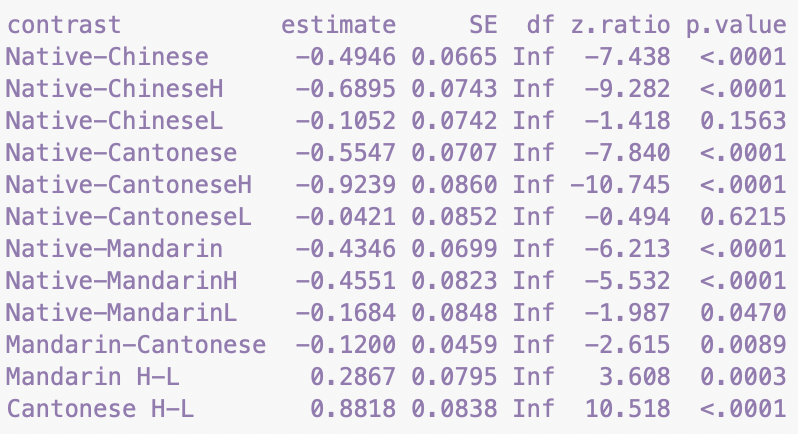 |

| **Experienced ~ Talker + (Talker \| ID)** |
| --- |
| 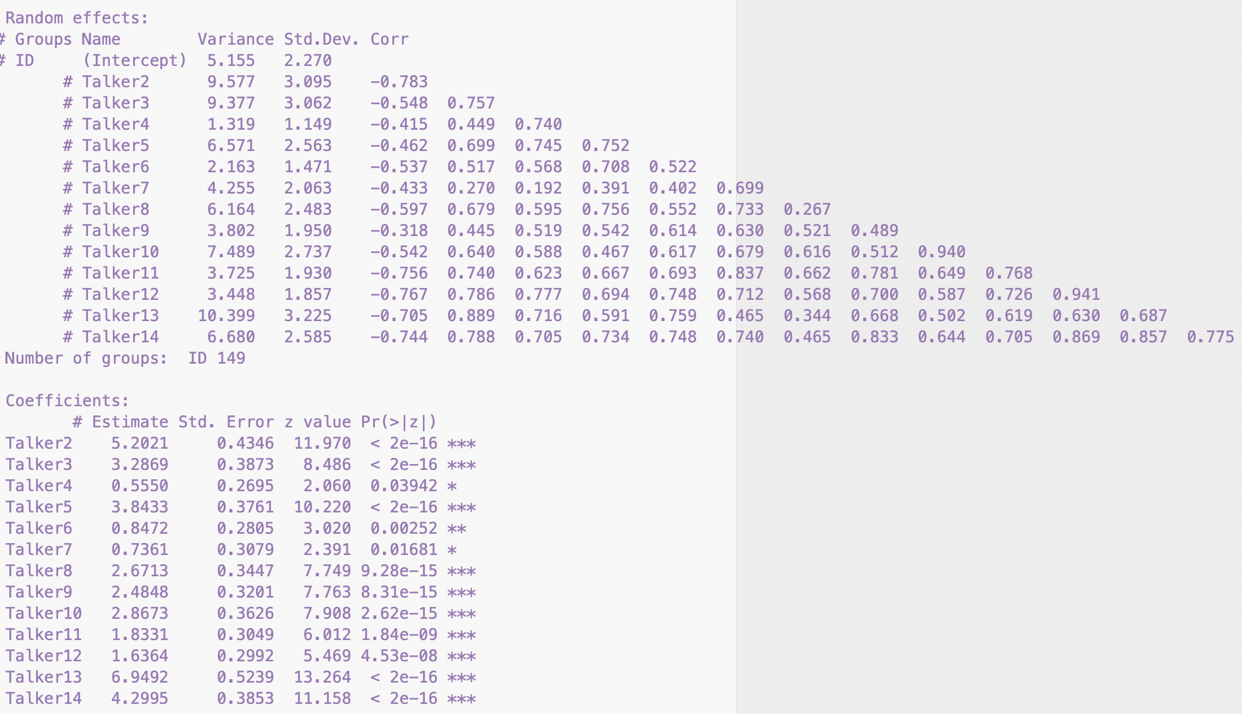  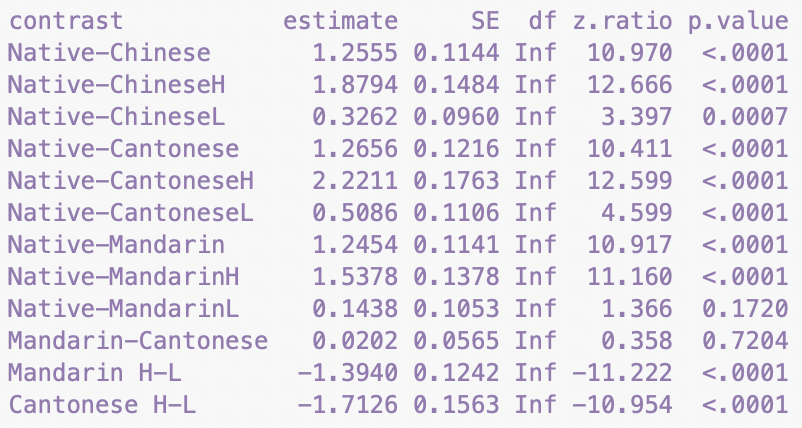 |

| **Friendly ~ Talker + (Talker \| ID)** |
| --- |
| 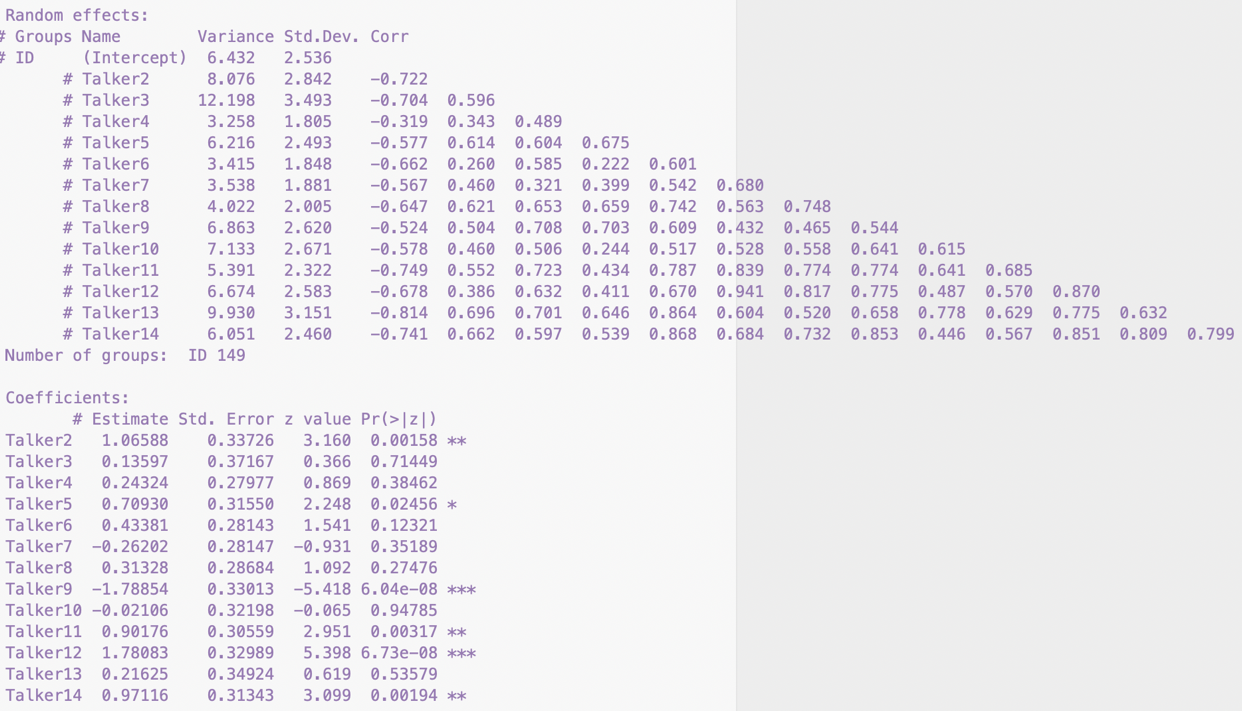  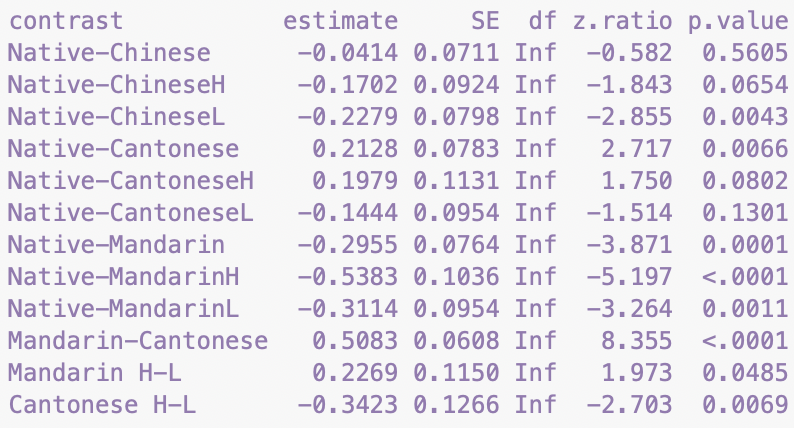 |

| *** Arrogant ~ Talker + (Talker \| ID)** |
| --- |
| 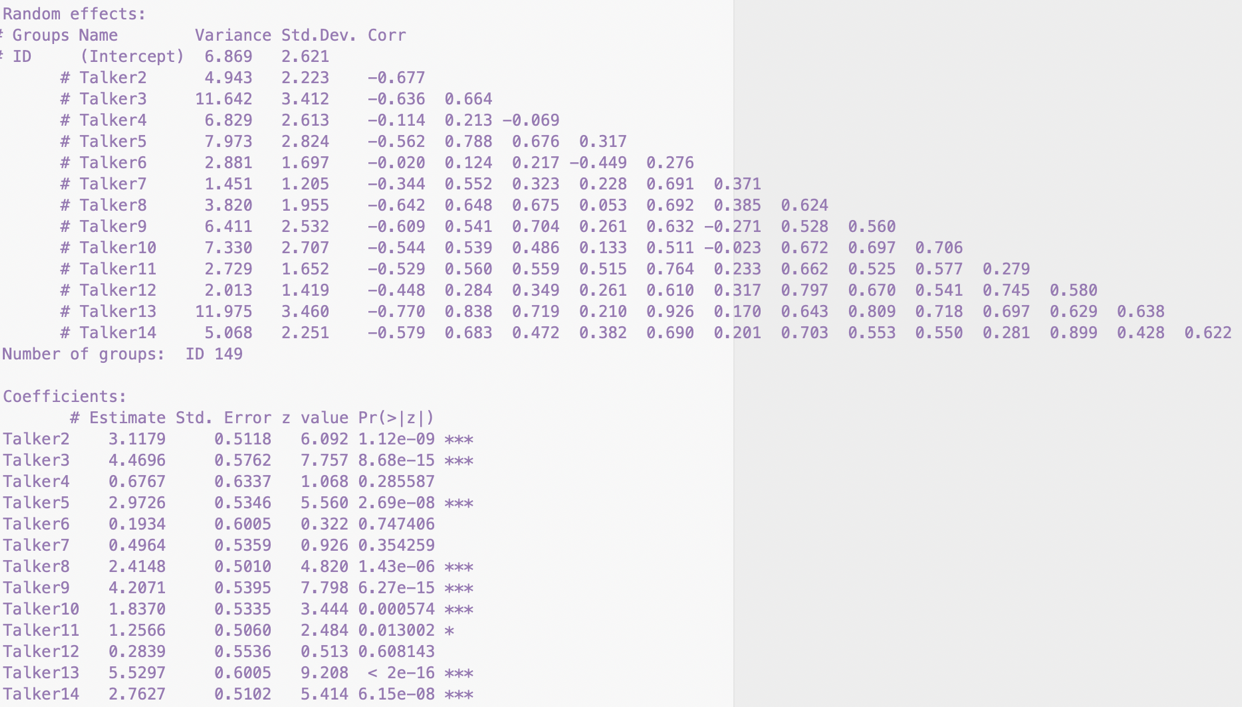  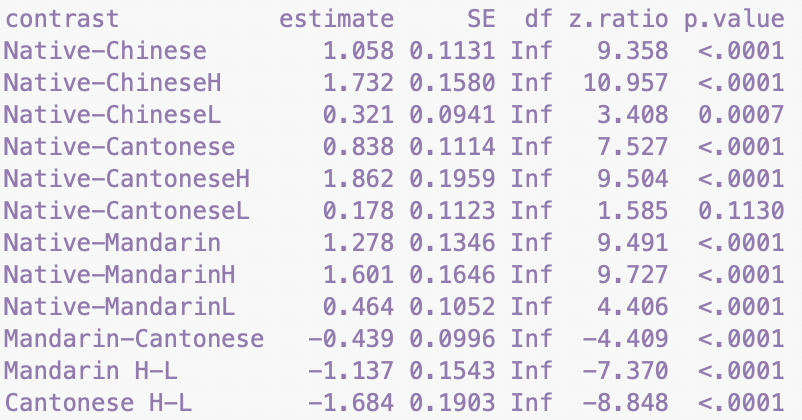 |

| **Sincere ~ Talker + AcqOrder + (Talker \| ID)** |
| --- |
| 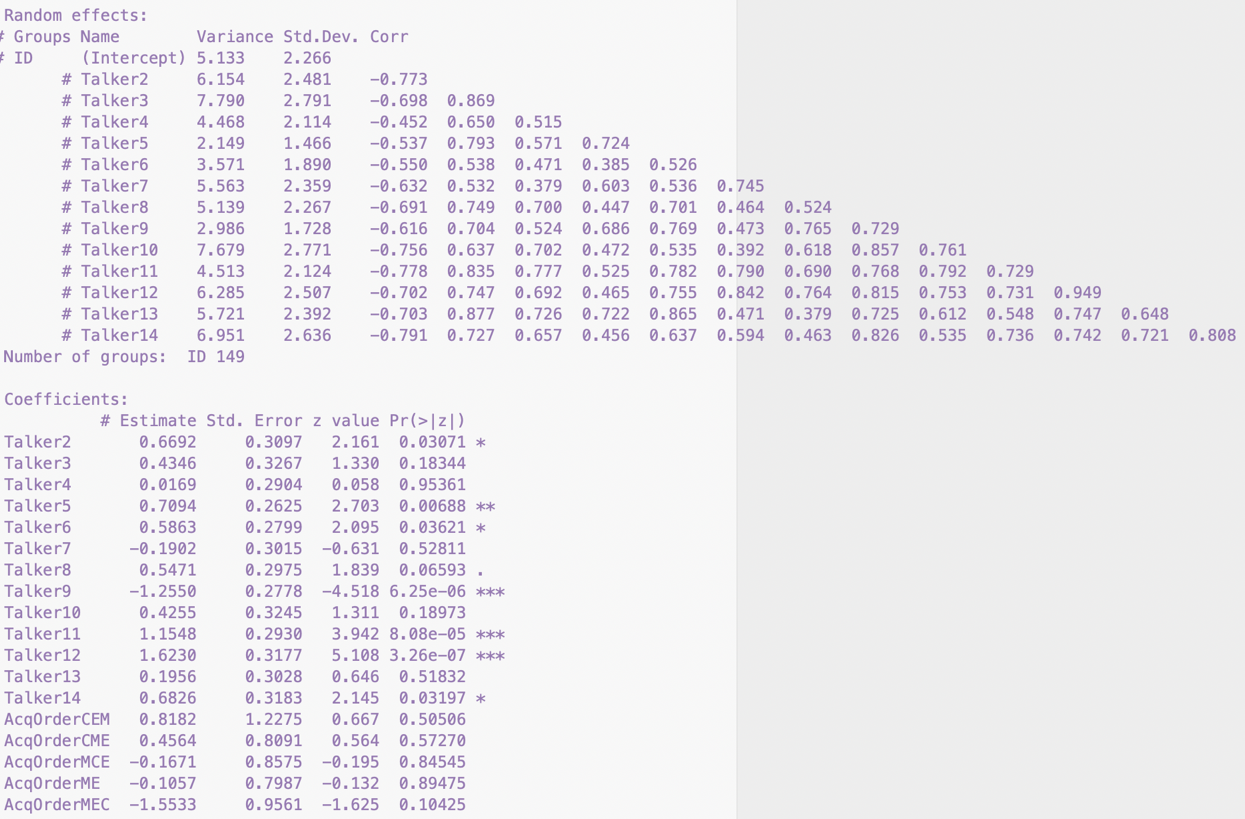  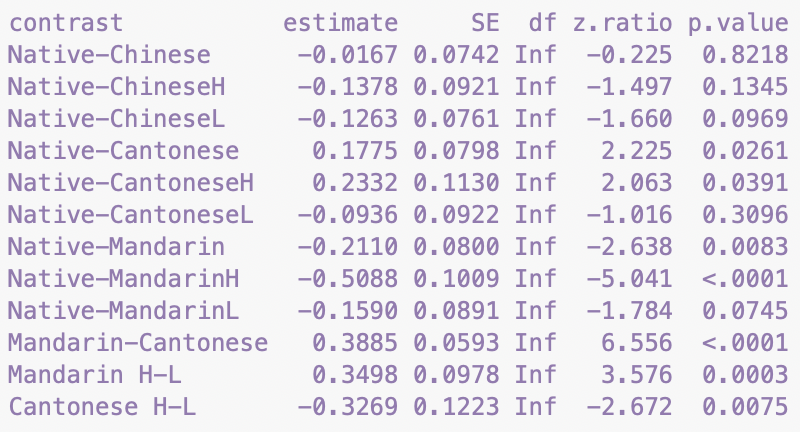 |

| **Approachable ~ AcqOrder + Talker + (Talker \| ID)** |
| --- |
| 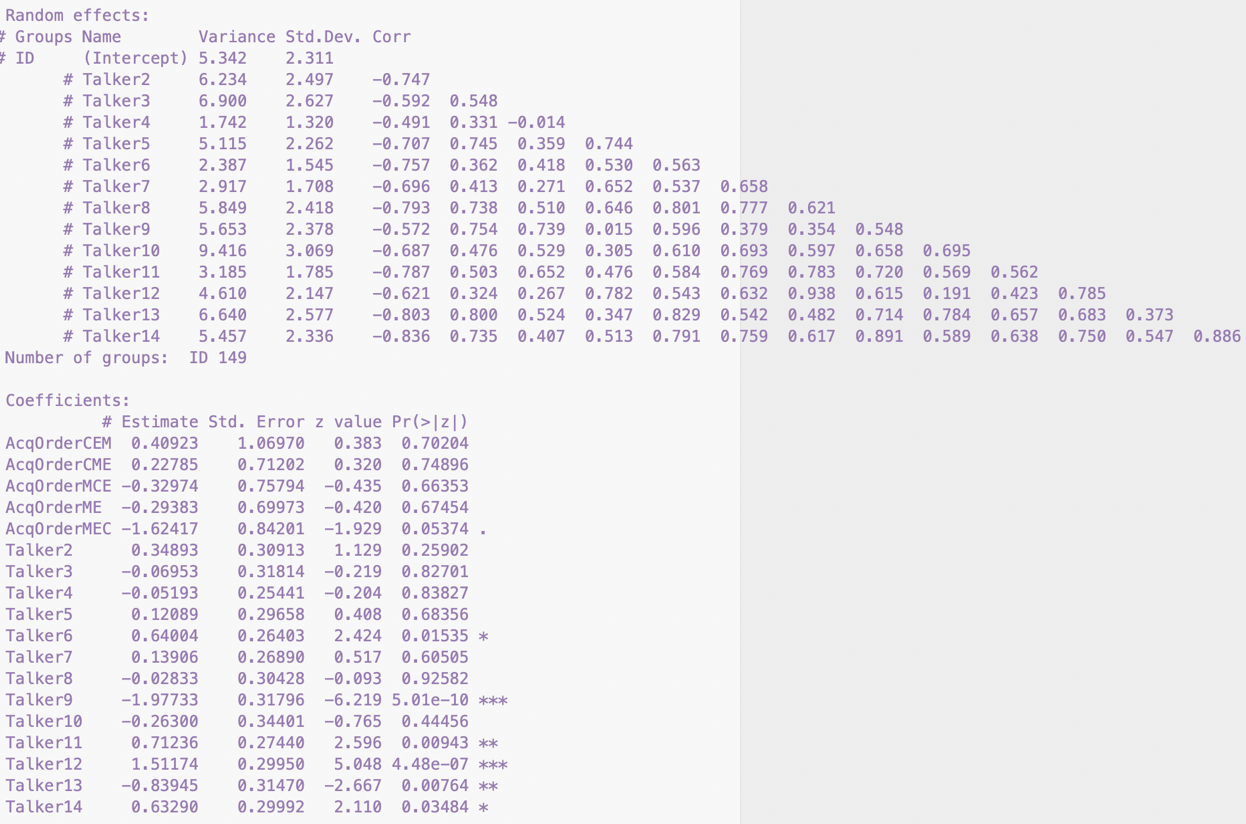  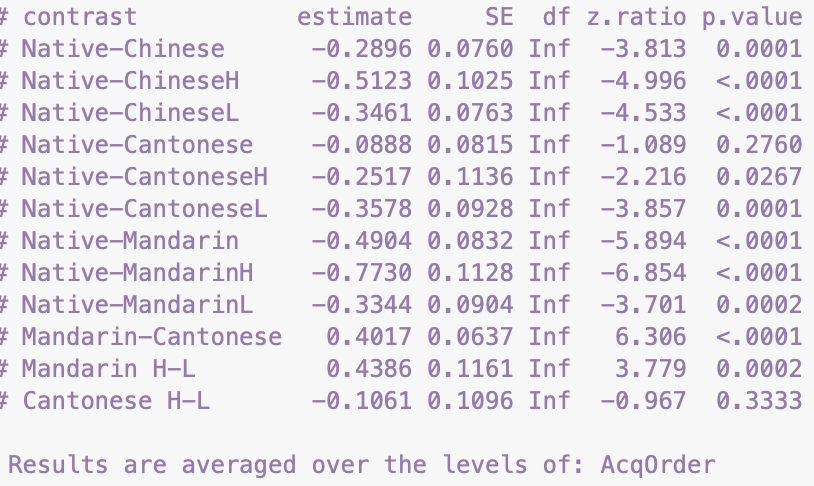 |

| **Considerate ~ Talker + (Talker \| ID)** |
| --- |
| 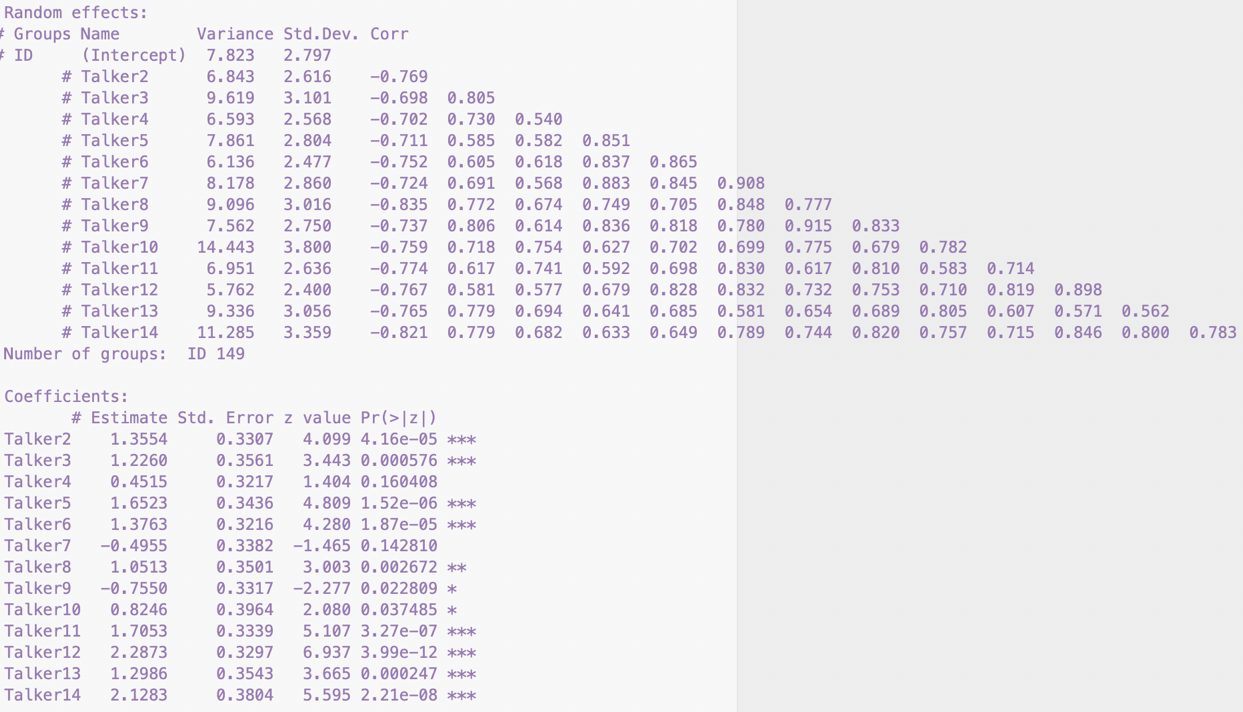  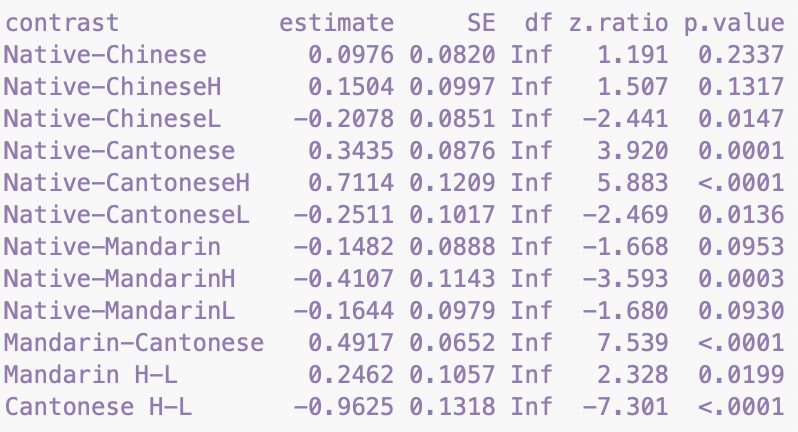 |

| **Trustworthy ~ Talker + (Talker \| ID)** |
| --- |
| 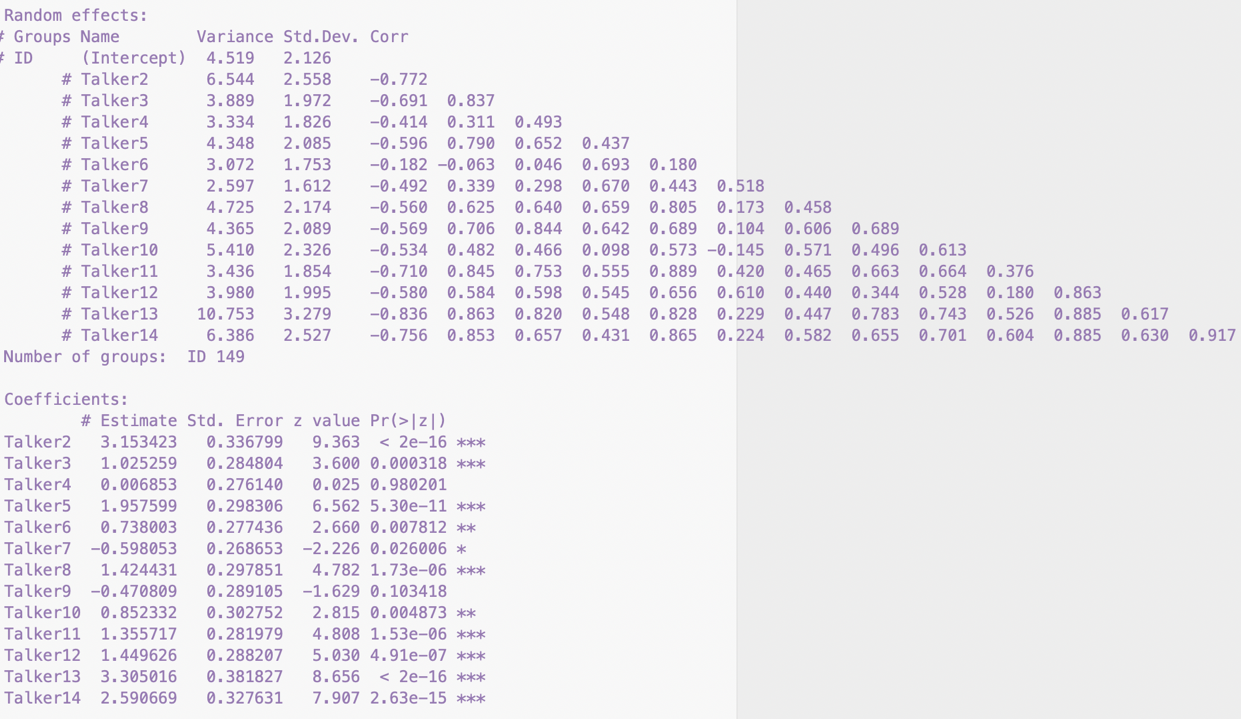  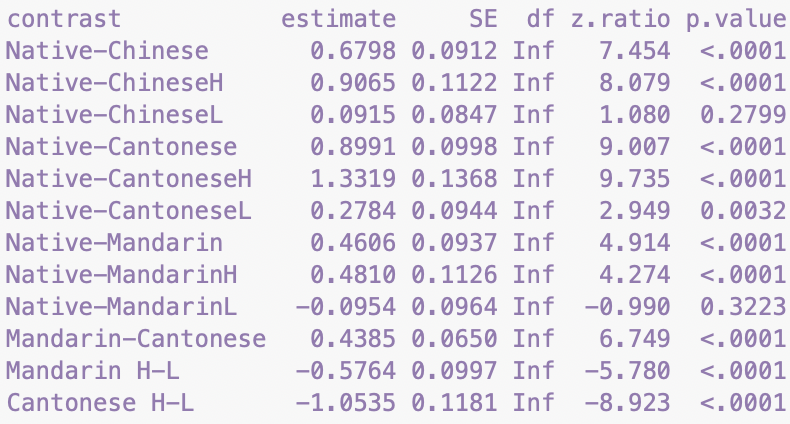 |

| **Industrious ~ Talker + (Talker \| ID)** |
| --- |
| 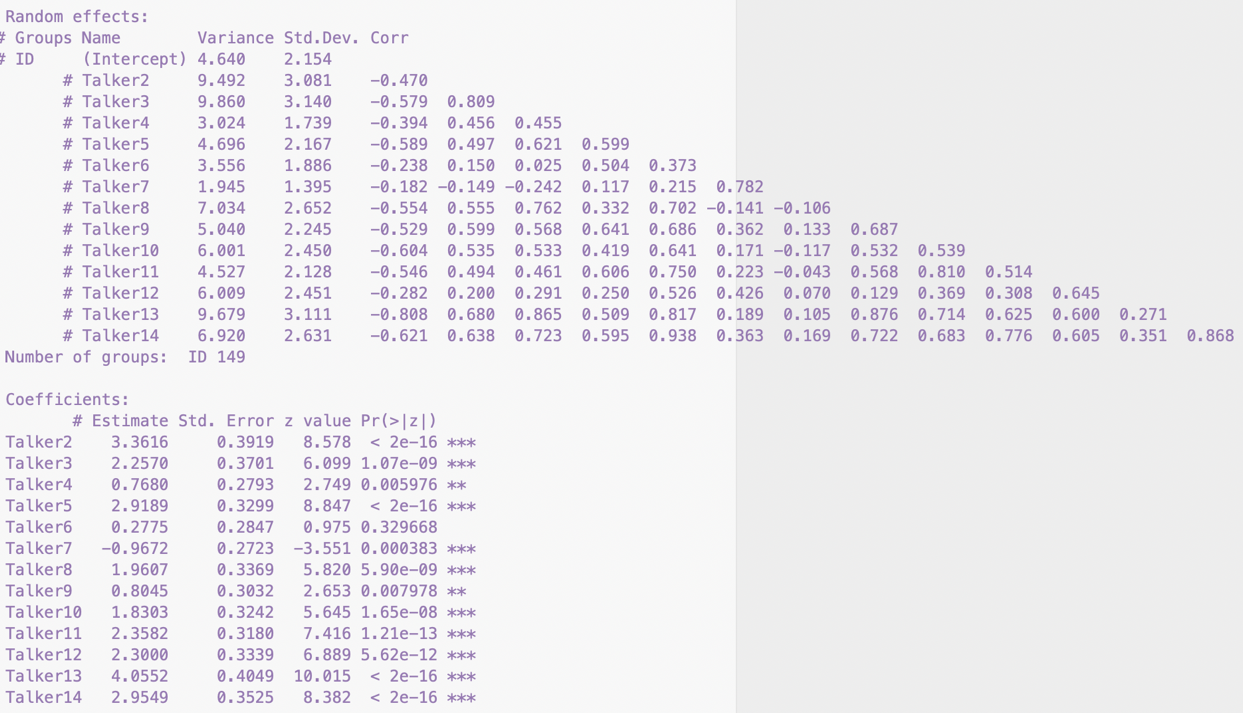  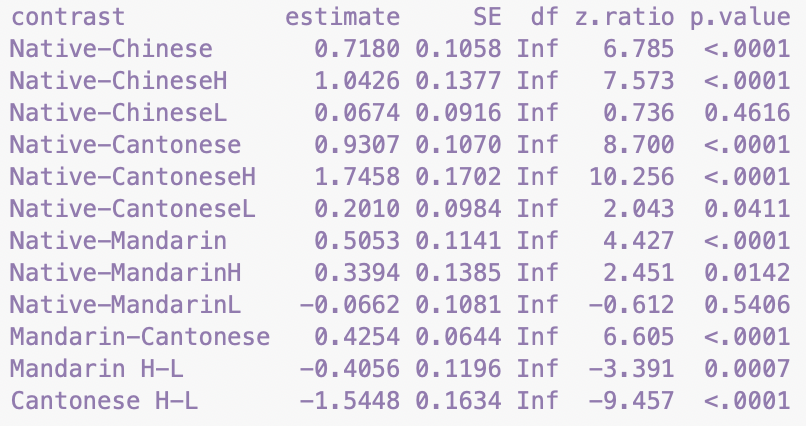 |

| *** Aggressive ~ Talker + (1 \| ID) + Sex** |
| --- |
| 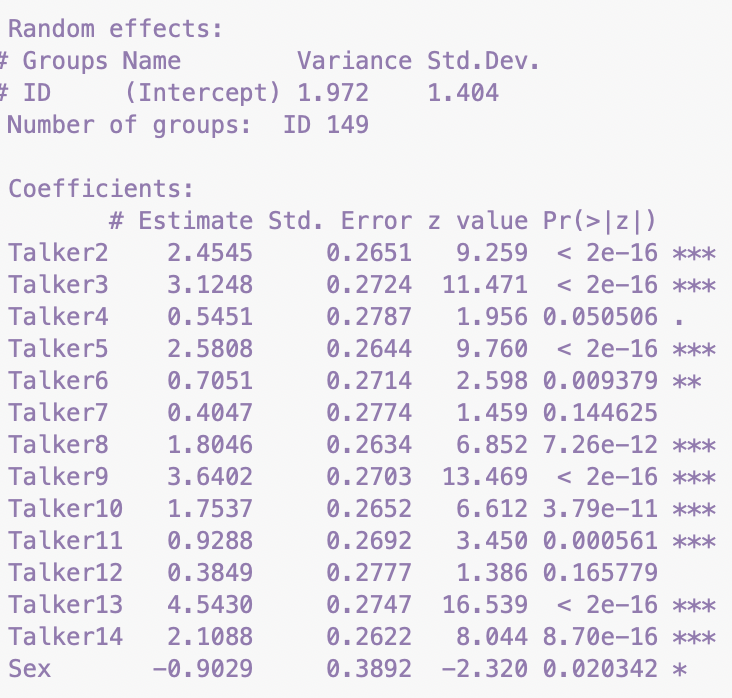  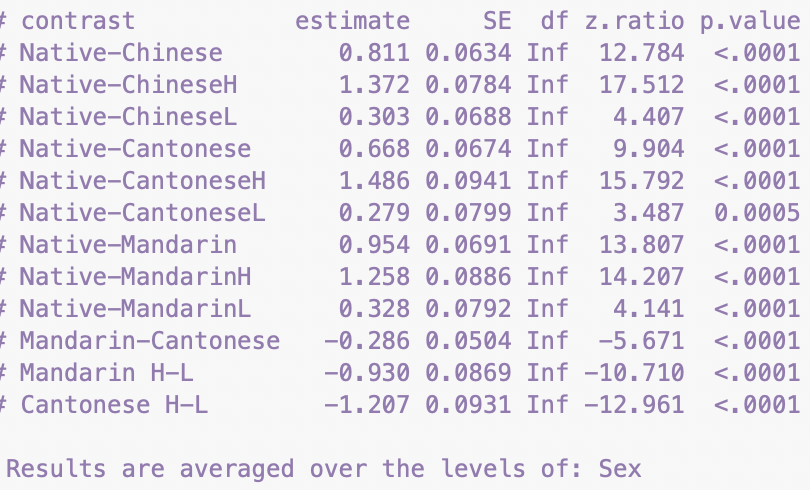 |

| **Trendy ~ (1 \| ID) + Talker + Sex** |
| --- |
| 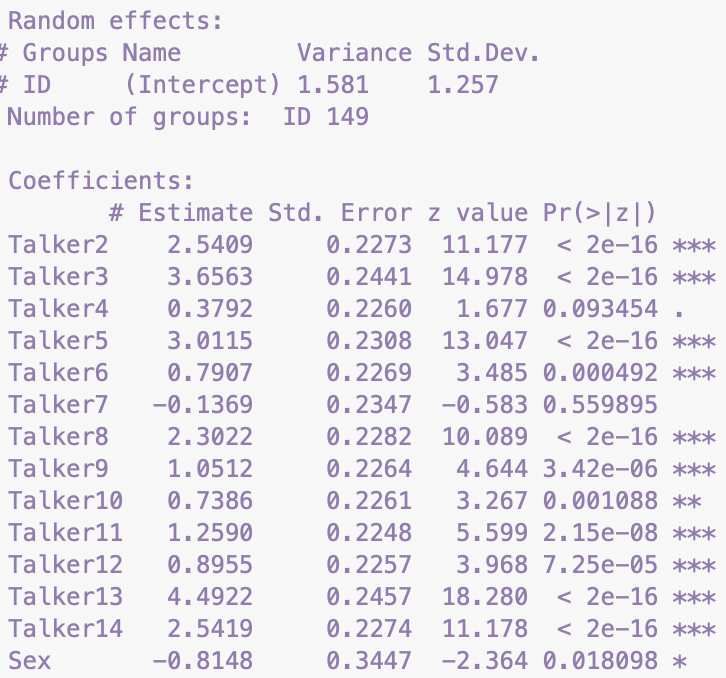 |

| *** Passive ~ Talker + (Talker \| ID)** |
| --- |
| 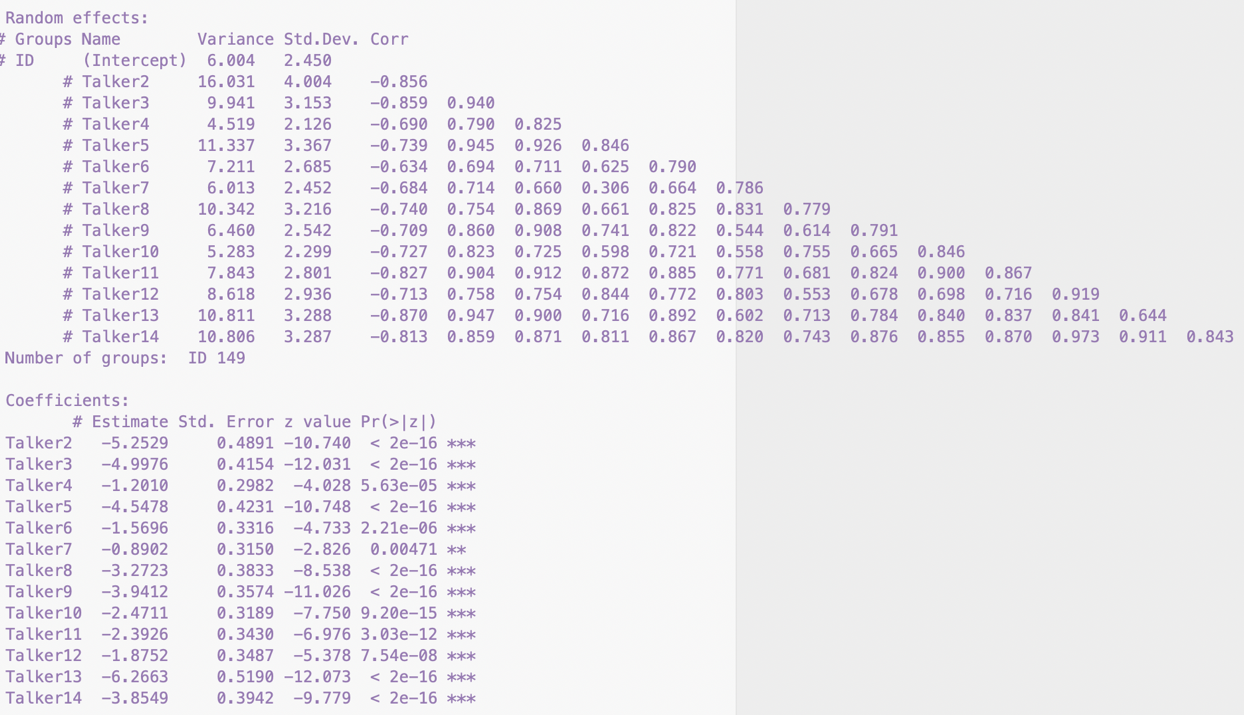  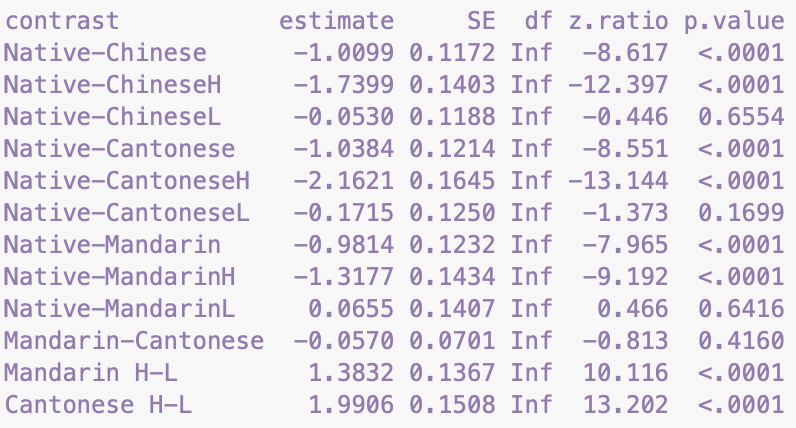 |

| *** Shy ~ Talker + (1 \| ID)** |
| --- |
| 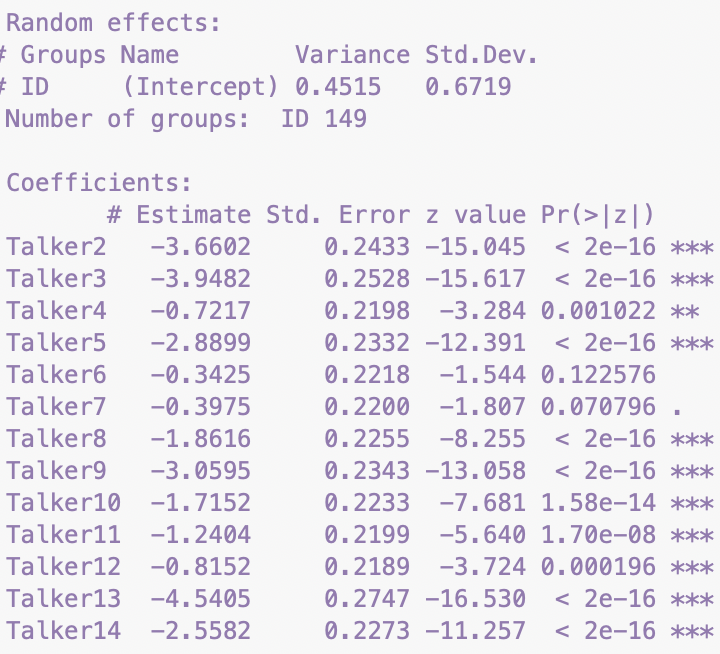  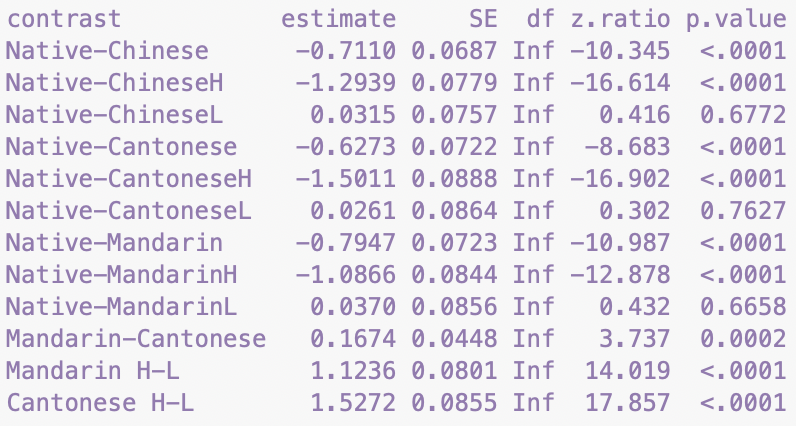 |

| **Confident ~ Talker + (Talker \| ID)** |
| --- |
| 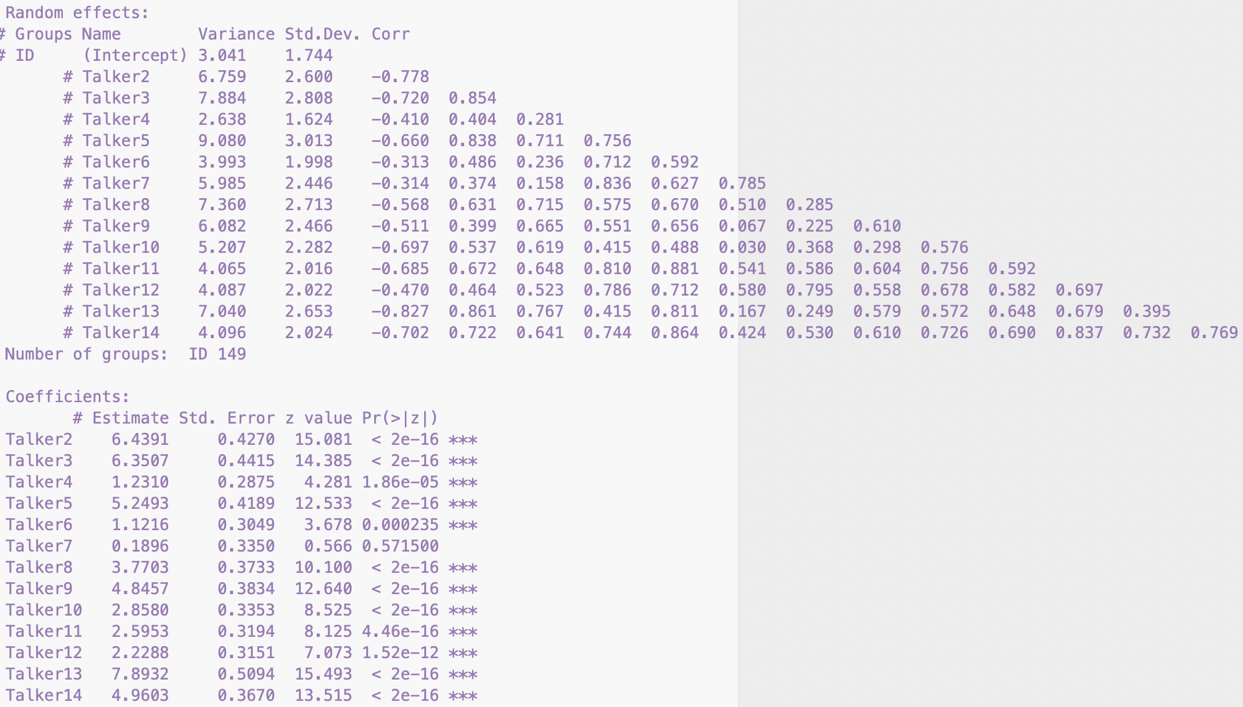  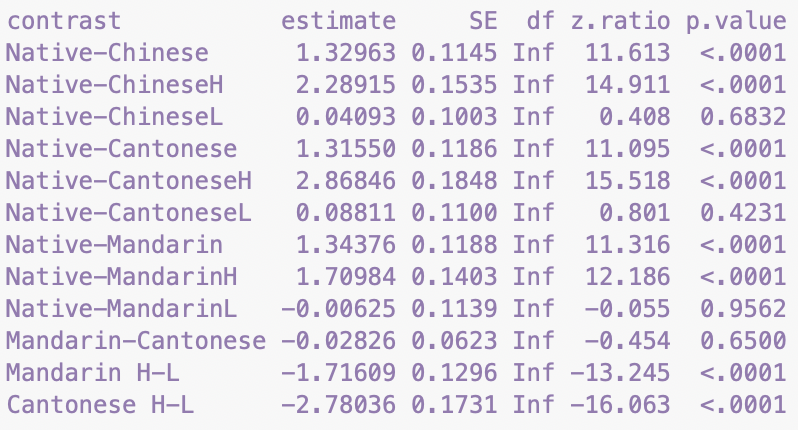 |
